# A procedure for maize genotypes discrimination to drought by chlorophyll fluorescence imaging rapid light curves

**DOI:** 10.1186/s13007-017-0209-z

**Published:** 2017-07-26

**Authors:** Carlos Antônio Ferreira de Sousa, Dayane Silva de Paiva, Raphael Augusto das Chagas Noqueli Casari, Nelson Geraldo de Oliveira, Hugo Bruno Correa Molinari, Adilson Kenji Kobayashi, Paulo Cesar Magalhães, Reinaldo Lúcio Gomide, Manoel Teixeira Souza

**Affiliations:** 1 Embrapa Agroenergia, Parque Estação Biológica (PqEB), Avenida W3 Norte (Final), Brasília, DF 70770-901 Brazil; 2 Embrapa Milho e Sorgo, Rod. MG 424 km 45, Zona Rural, Sete Lagoas, MG 35701-970 Brazil

**Keywords:** Corn, *Zea mays* L., Water deficit, Abiotic stress, Phenotyping, Gas exchange, Phenomics

## Abstract

**Background:**

Photosynthesis can be roughly separated into biochemical and photochemical processes. Both are affected by drought and can be assessed by non-invasive standard methods. Gas exchange, which mainly assesses the first process, has well-defined protocols. It is considered a standard method for evaluation of plant responses to drought. Under such stress, assessment of photochemical apparatus by chlorophyll fluorescence needs improvement to become faster and reproducible, especially in growing plants under field conditions. For this, we developed a protocol based on chlorophyll fluorescence imaging, using a rapid light curve approach.

**Results:**

Almost all parameters obtained by rapid light curves have shown statistical differences between control and drought stressed maize plants. However, most of them were affected by induction processes, relaxation rate, and/or differences in chlorophyll content; while they all were influenced by actinic light intensity on each light step of light curve. Only the normalized parameters related to photochemical and non-photochemical quenching were strongly correlated with data obtained by gas exchange, but only from the light step in which the linear electron flow reached saturation.

**Conclusions:**

The procedure developed in this study for discrimination of plant responses to water deficit stress proved to be as fast, efficient and reliable as the standard technique of gas exchange in order to discriminate the responses of maize genotypes to drought. However, unlike that, there is no need to perform daily and time consuming calibration routines. Moreover, plant acclimation to the dark is not required. The protocol can be applied to plants growing in both controlled conditions and full sunlight in the field. In addition, it generates parameters in a fast and accurate measurement process, which enables evaluating several plants in a short period of time.

**Electronic supplementary material:**

The online version of this article (doi:10.1186/s13007-017-0209-z) contains supplementary material, which is available to authorized users.

## Background

In recent years, it has been reported an increase in the frequency and intensity of extreme weather events such as cold, heat, flooding and drought especially in those regions with intensive plant cultivation and large production of food in the world [1, 2]. Such adverse weather events have potential to greatly reduce the crops productivity and, consequently, jeopardize the food supply worldwide [3]. Whenever the environmental variables undergo changes, moving away from a range considered ideal for plant cultivation towards an extreme limit, they creates the conditions for the occurrence of what is known as abiotic stresses [4]. Abiotic stresses can be defined as environmental conditions that reduce growth and yield below optimum levels [5]. Currently, the water availability in the soil is the variable that contributes the most to stress by drought, among others environmental variables with the greatest potential to cause stress in plants [6].

In order to deal with any stressful situation, plants trigger several mechanisms that work in all the levels of organization [7]. Under drought, it is widely accepted that one of the primary effects on plants is a reduction on the stomatal opening, with a consequent decrease in the water vapor and CO_2_ conductance [8, 9]. This mechanism reduces the water vapor loss, but, as a side effect, it also restricts CO_2_ entry in the leaf mesophyll [10]. Therefore, the measurement of gas exchange is considered a standard technique for studies related to this kind of stress. Consequently, the variables derived from the measurement of gas exchange, especially CO_2_ assimilation rate, stomata conductance and transpiration have been the most used to discriminate the plant responses to stress by water deficit [11].

Despite the fact that its initial manifestation is mainly perceived on gas exchange, stress by water deficit is one of the most comprehensive stresses regarding the effects on plant metabolism. It results from the fact that, in general, water shortage itself affects several processes, and it is usually associated with an increase in temperature and light intensity [12], enhancing the effects of drought mainly in tropical environments. Consequently, parallel to the effect on the gas exchange and the photosynthetic metabolism, damages to the structures and consequently to the photochemical processes occurring inside the chloroplasts are enhanced [13].

Drought not only causes degradation of photosynthetic pigments, but also disorganization of the thylakoid membranes [13, 14]. In such membranes are anchored the components of the primary photochemistry of photosynthesis, represented mainly by the light-harvesting complexes and their respective reaction centers of photosystems I and II [15]. The latter is responsible for using the energy of the absorbed light to drive the electron flow through the chloroplast membrane system [16–18].

The loss of pigments reduces the absorption of light energy and, in the first instance, may even attenuate the potential damages caused by the drought on the photochemical apparatus [19]. However, the disorganization of the thylakoid membranes causes damage to the reaction centers of photosystem II [13], which itself impairs the primary reactions of light energy conversion to chemical energy [20]. Due to the restriction of CO_2_ entry in chloroplasts during drought, there is a decrease in primary photochemical processes [20]. In order to avoid damage to the photochemical apparatus, the captured light energy is partially diverted to different processes [21–23].

The light energy absorbed by chlorophylls associated with PSII can be used to drive photochemistry, lost from PSII as heat or emitted as chlorophyll fluorescence [13, 14, 17, 24, 25]. The chlorophyll fluorescence parameters that can be used to estimate the flux of excitation energy into three competing pathways are Y(II), Y(NPQ) and Y(NO) [26]. In the first two cases the emission of fluorescence does not occur, that is, it is quenched, and the resulting processes are called photochemical and non-photochemical quenching of chlorophyll fluorescence, respectively. Separation of fluorescence quenching into photochemical and non-photochemical components can be reached by chlorophyll fluorescence technique, using the saturation pulse method [17]. This method basically measures the fluorescence signal emitted under dark (Fo, Fm) or actinic illumination (Fs, Fm′) [16].

From the saturation pulse method, and considering the model of antenna pigment organization in the thylakoids as a puddle or a lake [18], several parameters were developed which correlate with photochemical and non-photochemical quenching [26, 27]. Thus, Y(II), qP, and Fv/Fm are considered as photochemical quenching parameters, while NPQ is non-photochemical [28]. qL and qN represent respectively the photochemical and non-photochemical quenching in the lake model, while qP represents photochemical quenching in the puddle model [26]. For all this, chlorophyll fluorescence is considered as a probe of the photochemical apparatus [25]. In addition, it has been proven to be an efficient and reproducible method for evaluating plants under stress [29].

The measurements obtained by the chlorophyll fluorescence technique can be rapid, highly sensitive and non-invasive [17, 25, 30]. They can be performed on intact leaves still attached to plants. This technique has been used to discriminate the responses to drought in controlled conditions mainly from induction curves [31–33] since the simple measurement of Fv/Fm seems to work only in cases of severe drought [34, 35]. However, to evaluate a large number of plants under real environmental conditions in the field, induction curves (IC) would not be the most recommended approach. This is because IC is a procedure in which plant photosynthetic apparatus, after a previous period of dark adaptation, sufficient to completely oxidize the PSII reaction center, is re-submitted to illumination [36]. The protocols currently employed for IC’s running allows the correct measurement of Fo and Fm, which are fundamental for the calculation of Fv/Fm and all parameters related to photochemical and non-photochemical fluorescence quenching [17, 25]. However, IC’s require a prior period of darkness of at least 15 min and the measurement process itself takes longer, i.e., about 5 min per sample. To overcome these challenges, especially when using chlorophyll fluorescence technology for generating images of increasingly larger areas in high throughput systems, an increasing trend nowadays [37–39], researchers have used new approaches that work very well on model plants under highly controlled conditions [40, 41]. Nonetheless, such approaches do not work for large plants grown in the field. In this case, to get a fast image of chlorophyll fluorescence parameters, rapid light curves (RLC’s) can be considered as an option.

RLC’s capture the instantaneous responses of plants in a considerable range of light intensity by the simultaneous evaluation of a number of parameters. The biggest advantage of this approach is that it does not demand acclimatization to dark. Moreover, it can be applied to plants grown in full sunlight which saves time and increases the measuring range. However, if the RLC’s are performed without pre-dark acclimation, the initial fluorescence measurements obtained do not correspond to the theoretical values of Fo and Fm. Consequently, the calculated fluorescence quenching does not match to actual values. Therefore, RLC’s are usually used to determine the maximum electron transport rate as well as other parameters [42, 43]. Nevertheless, if the aim of the study does not depend on an accurate measure of Fo, Fm and photochemical and non-photochemical quenching, such parameters obtained from RLC’s without prior dark-acclimation can be used [43]. Previous studies using RLC’s approach for discrimination of plant responses to stresses only compared chlorophyll fluorescence parameters obtained along the light curve [13, 42, 43].

In this study, we used adult maize plants of different genotypes grown under field conditions which were subjected to drought in the pre-flowering stage. Since the cultivation conditions restricted the approaches options for evaluation by chlorophyll fluorescence, we applied upward RLC’s on plants without pre-dark-acclimation to discriminate the genotype responses to drought. The RLC’s were dissected in its components light steps and the measured chlorophyll fluorescence parameters in each of them were correlated with gas exchange. Based on the induction effects of the light curve itself and the effects of drought on the photochemical apparatus of plants, we argued that not all measured chlorophyll fluorescence parameters nor all light steps can be used for correct discrimination of maize genotypes to drought. Only normalized photochemical and non-photochemical parameters, measured in the light step in which the linear electron flow reached saturation, can be indicated for this. All of them were as efficient and reliable as the standard technique of gas exchange in order to discriminate the responses of maize genotypes to stress by water deficit. From this study, it was proposed a procedure to be used for this aim, which standardizes the type and portion of the leaf to be evaluated, the RLC’s parameters configuration and the ideal light step in which the most appropriate parameters potentiate the differences.

## Results

### Changes in chlorophyll fluorescence parameters over RLC’s and chlorophyll content in control and drought stressed maize plants

**Fig. 1:**
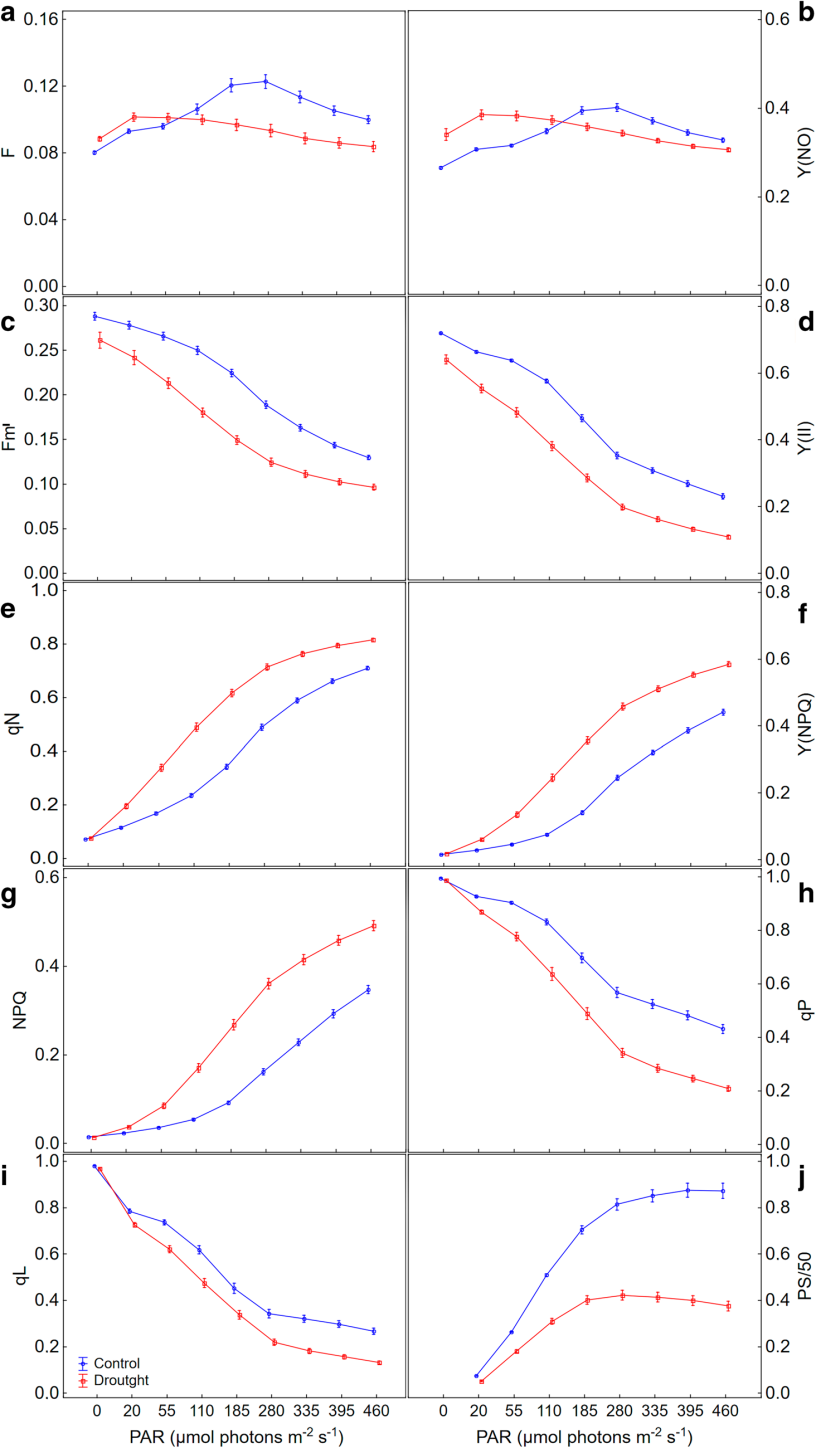
Chlorophyll fluorescence parameters generated by rapid light curves applied to leaves for control and drought stressed maize plants as a function of photosynthetically active radiation. **a** F, fluorescence yield; **b** Y(NO), quantum yield of nonregulated energy dissipation; **c** Fm′, maximum fluorescence yield on light-adapted leaf; **d** Y(II), effective PSII quantum yield; **e** qN, coefficient of non-photochemical quenching (lake model); **f** Y(NPQ), quantum yield of regulated energy dissipation; **g** NPQ, non-photochemical quenching; **h** qP, coefficient of photochemical quenching (puddle model); **i** qL, coefficient of photochemical quenching (lake model); **j** PS/50, apparent rate of photosynthesis. All maize plants were grown with soil water content at field capacity. At the V16 stage a group of plants of each genotype was subjected to water withholding until reach the theoretical wilting point (drought) and remaining for 12 days while another group was kept under field capacity (control). The value of each parameter in every light step represents the average of four measurements over the period of stress in all maize genotypes studied under control or drought stress. *Bars* represent standard error of the mean

All chlorophyll fluorescence parameters obtained throughout the RLC’s showed statistically significant differences between control and drought stressed maize plants. For most parameters, the biggest differences between stressed and not stressed plants occurred from the middle to the end portion of the RLC’s, except for the fluorescence yield (F) and for the quantum yield of nonregulated energy dissipation [Y(NO)] (Fig. 1; Additional files 1, 2).

The RLC’s relative to F and Y(NO) showed similar shape for stressed and non-stressed plants (Fig. 1a, b). The means of these two parameters, measured without actinic illumination (light step zero), were higher in plants subjected to drought in comparison to control. After starting actinic illumination and a gradual increase in light levels, both parameters increased for both groups of plants. However, the rises occurred only in the first step of actinic light in drought stressed plants and up to the fourth light step in the control ones. Then, F and Y(NO) began to decline in a faster and more intense manner in plants under stress. At the end of the RLC’s, the means of both parameters in stressed plants become smaller than those of control plants.

The RLC’s representing parameters known as maximum fluorescence yield in the light (Fm′) and effective PSII quantum yield [Y(II)] also showed a very similar pattern to each other (Fig. 1c, d). Both parameters showed an expected trend of decreasing the extent to which actinic light has increased. However, the control plants showed higher Fm′ and Y(II) means throughout all the RLC’s steps. Similarly to what had happened to F and Y(NO), the decline in Fm′ and Y(II) means was much higher and faster in plants subjected to drought stress compared to the control ones. The major differences in Fm′ and Y(II) means between stressed and control plants occurred at the intermediate portion of the RLC’s, whereas in extreme ends there was a tendency of shortening of these differences.

The parameters representing regulated dissipation of light energy as heat [qN, Y(NPQ) and NPQ] (Fig. 1e–g) increased along the RLC’s for both groups of maize plants, stressed and non-stressed. Nevertheless, the increase was faster and more robust on drought stressed plants, so that the means of all those parameters remained higher in this group of plants compared to controls. On the other hand, the parameters related to photochemical dissipation of light energy [Y(II), qP and qL] (Fig. 1d, h, i) decreased. For these parameters, the decrease was more pronounced in stressed plants.

Instead of ETR, we measured a similar parameter called the apparent rate of photosynthesis (PS). While for ETR a PAR-absorptivity of 0.84 is assumed, for PS the PAR-absorptivity is determined using an IMAGING-PAM routine. Calculated in this way, the PS values reached around 50. Then, to allow them to be visualized by the color bar, whose range varies from 0 to 1, the PS values were divided by 50, hence PS/50. As expected, the apparent rate of photosynthesis (PS/50) went up with the increase in the intensity of the actinic light up to saturation level. The treatments by which the plants were subjected have influenced the intensity and speed of the PS/50 increase as well as the actinic light intensity in which saturation occurred (Fig. 1j). It was observed that, in stressed plants, PS/50 increased to a less extent degree and saturation occurred at a lower light level, around 185 µmol m^−2^ s^−1^, while on the control plants it increased in a more pronounced way and saturation occurred at 280 µmol m^−2^ s^−1^.

**Fig. 2:**
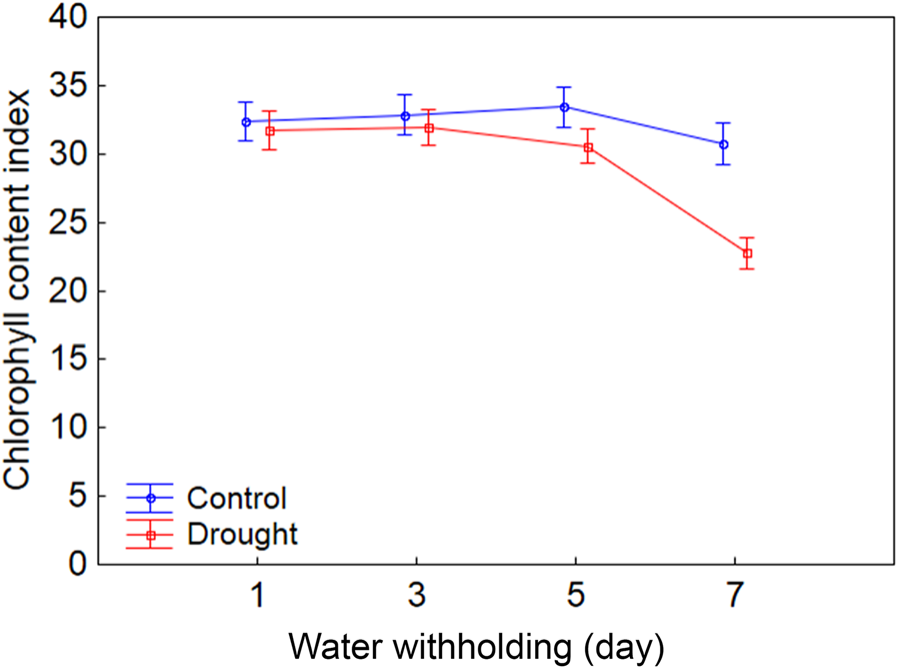
Changes over time in chlorophyll content index for control and drought stressed maize plants. The values of the parameters obtained at every 2 days represent the average of 20 replicates considering all studied maize genotypes. Both groups of plants (control and drought) were held with soil water content at field capacity at the start of measurement (±60 days after sowing; 1st day). From there, the watering was withheld in the drought stressed plants until the substrate reached the theoretical wilting point (−1.5 MPa) on the 7th day. *Bars* represent standard error of the mean

Since the non-normalized chlorophyll fluorescence parameters, such as F and Fm′, are affected by leaf chlorophyll content, the chlorophyll content index (CCI) was measured in the leaves of control and drought stressed maize plants throughout the experiment. This parameter did not change in the leaves of control plants over the experimental period, while it was observed a fall in drought stressed plants (Fig. 2; Additional file 3).

### Relationship among RLC’s chlorophyll fluorescence parameters and gas exchange

Most of the parameters obtained from every RLC’s light step showed statistically significant correlation with those of gas exchange (Table 1). However, in the initial steps of actinic lighting, the correlation coefficient for most parameters lies in the range considered as from moderate to weak. As the intensity of the actinic light increased, it was seen an increase in the correlation coefficient until 185 µmol m^−2^ s^−1^ for most parameters, except for F and Y(NO). For these parameters, the correlation decreased from the initial to the middle portion of the light curve and increased again afterward.

**Table 1:** Correlation coefficients among gas exchange and chlorophyll fluorescence parameters derived at each light step of rapid light curves

PAR	GEM	Chlorophyll fluorescence parameter									
		F	Fm′	Y(II)	Y(NPQ)	Y(NO)	NPQ	qN	qP	qL	PS/50
0	*A*	−0.52**	0.28^ns^	0.58**	−0.62**	−0.57**	0.33*	−0.19^ns^	0.50**	0.53**	
	*gs*	−0.52**	0.22^ns^	0.53**	−0.59**	−0.52**	0.30^ns^	−0.22^ns^	0.44**	0.47**	
	*Ci*	0.25^ns^	−0.29^ns^	−0.49**	0.36*	0.49**	−0.35*	−0.10^ns^	−0.50**	−0.51**	
	*E*	−0.51**	0.28^ns^	0.58**	−0.62**	−0.57**	0.34*	−0.20^ns^	0.49**	0.52**	
20	*A*	−0.47**	0.38*	0.71**	−0.73**	−0.66**	−0.63**	−0.68**	0.76**	0.60**	0.76**
	*gs*	−0.49**	0.31*	0.66**	−0.67**	−0.62**	−0.56**	−0.60**	0.73**	0.61**	0.72**
	*Ci*	0.22^ns^	−0.37*	−0.56**	0.56**	0.52**	0.47**	0.55**	−0.55**	−0.37**	−0.56**
	*E*	−0.46**	0.38*	0.71**	−0.73**	−0.66**	−0.62**	−0.67**	0.76**	0.59**	0.76**
55	*A*	−0.33*	0.58**	0.83**	−0.87**	−0.64**	−0.81**	−0.84**	0.80**	0.72**	0.85**
	*gs*	−0.38*	0.50**	0.78**	−0.81**	−0.60**	−0.74**	−0.76**	0.77**	0.73**	0.80**
	*Ci*	0.11^ns^	−0.50**	−0.61**	0.61**	0.50**	0.58**	0.62**	−0.59**	−0.44**	−0.60**
	*E*	−0.33*	0.57**	0.82**	−0.86**	−0.63**	−0.80**	−0.83**	0.79**	0.71**	0.84**
110	*A*	0.03^ns^	0.71**	0.90**	−0.93**	−0.43**	−0.88**	−0.89**	0.79**	0.66**	0.92**
	*gs*	−0.05^ns^	0.62**	0.87**	−0.87**	−0.45**	−0.82**	−0.82**	0.77**	0.68**	0.88**
	*Ci*	−0.16^ns^	−0.55**	−0.61**	0.61**	0.33*	0.57**	0.61**	−0.59**	−0.38*	−0.60**
	*E*	0.03^ns^	0.70**	0.89**	−0.92**	−0.43**	−0.87**	−0.88**	0.79**	0.66**	0.91**
185	*A*	0.34*	0.76**	0.90**	−0.95**	0.19^ns^	−0.93**	−0.90**	0.80**	0.58**	0.91**
	*gs*	0.24^ns^	0.68**	0.88**	−0.90**	0.09^ns^	−0.87**	−0.83**	0.80**	0.63**	0.90**
	*Ci*	−0.34*	−0.56**	−0.55**	0.60**	−0.16^ns^	0.54**	0.60**	−0.49**	−0.26^ns^	−0.54**
	*E*	0.32*	0.74**	0.89**	−0.94**	0.16^ns^	−0.91**	−0.88**	0.80**	0.59**	0.91**
280	*A*	0.44**	0.73**	0.91**	−0.94**	0.45**	−0.91**	−0.85**	0.85**	0.67**	0.92**
	*gs*	0.34*	0.65**	0.91**	−0.89**	0.35**	−0.85**	−0.78**	0.86**	0.71**	0.91**
	*Ci*	−0.42**	−0.55**	−0.49**	0.57**	−0.40**	0.54**	0.59**	−0.43**	−0.24^ns^	−0.49**
	*E*	0.42**	0.71**	0.91**	−0.92**	0.42**	−0.90**	−0.83**	0.85**	0.68**	0.91**
335	*A*	0.47**	0.71**	0.91**	−0.93**	0.50**	−0.89**	−0.83**	0.87**	0.73**	0.92**
	*gs*	0.38*	0.64**	0.90**	−0.89**	0.41**	−0.83**	−0.76**	0.87**	0.76**	0.91**
	*Ci*	−0.44**	−0.55**	−0.50**	0.58**	−0.43**	0.55**	0.59**	−0.43**	−0.26^ns^	−0.50**
	*E*	0.45**	0.70**	0.91**	−0.92**	0.47**	−0.87**	−0.81**	0.87**	0.74**	0.92**
395	*A*	0.46**	0.70**	0.90**	−0.92**	0.47**	−0.86**	−0.81**	0.87**	0.76**	0.91**
	*gs*	0.38*	0.63**	0.89**	−0.88**	0.39**	−0.80**	−0.75**	0.87**	0.79**	0.90**
	*Ci*	−0.43**	−0.53**	−0.48**	0.53**	−0.38**	0.52**	0.57**	−0.43**	−0.29^ns^	−0.48**
	*E*	0.44**	0.68**	0.89**	−0.90**	0.44**	−0.84**	−0.79**	0.87**	0.77**	0.90**
460	*A*	0.44**	0.68**	0.88**	−0.90**	0.43**	−0.83**	−0.81**	0.87**	0.78**	0.90**
	*gs*	0.36**	0.61**	0.87**	−0.86**	0.36*	−0.77**	−0.75**	0.86**	0.80**	0.89**
	*Ci*	−0.41**	−0.53**	−0.46**	0.51**	−0.36*	0.50**	0.57**	−0.41**	−0.28^ns^	−0.46**
	*E*	0.43**	0.67**	0.88**	−0.88**	0.40*	−0.81**	−0.79**	0.86**	0.78**	0.89**

Abbreviations: photosynthetically active radiation (PAR, µmol photons m^−2^ s^−1^); gas exchange measurements (GEM); net CO_2_ assimilation rate (*A*); stomatal conductance to water vapor (*gs*); intercellular CO_2_ concentration (*Ci*); transpiration rate (*E*); fluorescence yield (not necessarily in the steady-state) before application a saturate pulse (F); quantum yield of nonregulated energy dissipation [Y(NO)]; maximum fluorescence yield on light-adapted leaf (Fm′); effective PSII quantum yield [Y(II)]; coefficient of non-photochemical quenching (qN, lake model); quantum yield of regulated energy dissipation [Y(NPQ)]; non-photochemical quenching (NPQ); coefficient of photochemical quenching (qP, puddle model); coefficient of photochemical quenching (qL, lake model); apparent rate of photosynthesis (PS/50)

^ns,^*^,^** Not significantly different, significant at the 5 and 1% probability levels, respectively

Despite the level of statistical significance in all light steps, the low coefficient correlations for F and Y(NO), preponderantly, are considered as moderate to weak. On the other hand, Fm′, Y(II), PS/50, qP and qL as well as qN, NPQ and Y(NPQ) showed strong correlations with the parameters of gas exchange, except *Ci*. On average, the correlation coefficient for Y(II) and its derived PS/50 were slightly higher than for the other parameters.

**Fig. 3:**
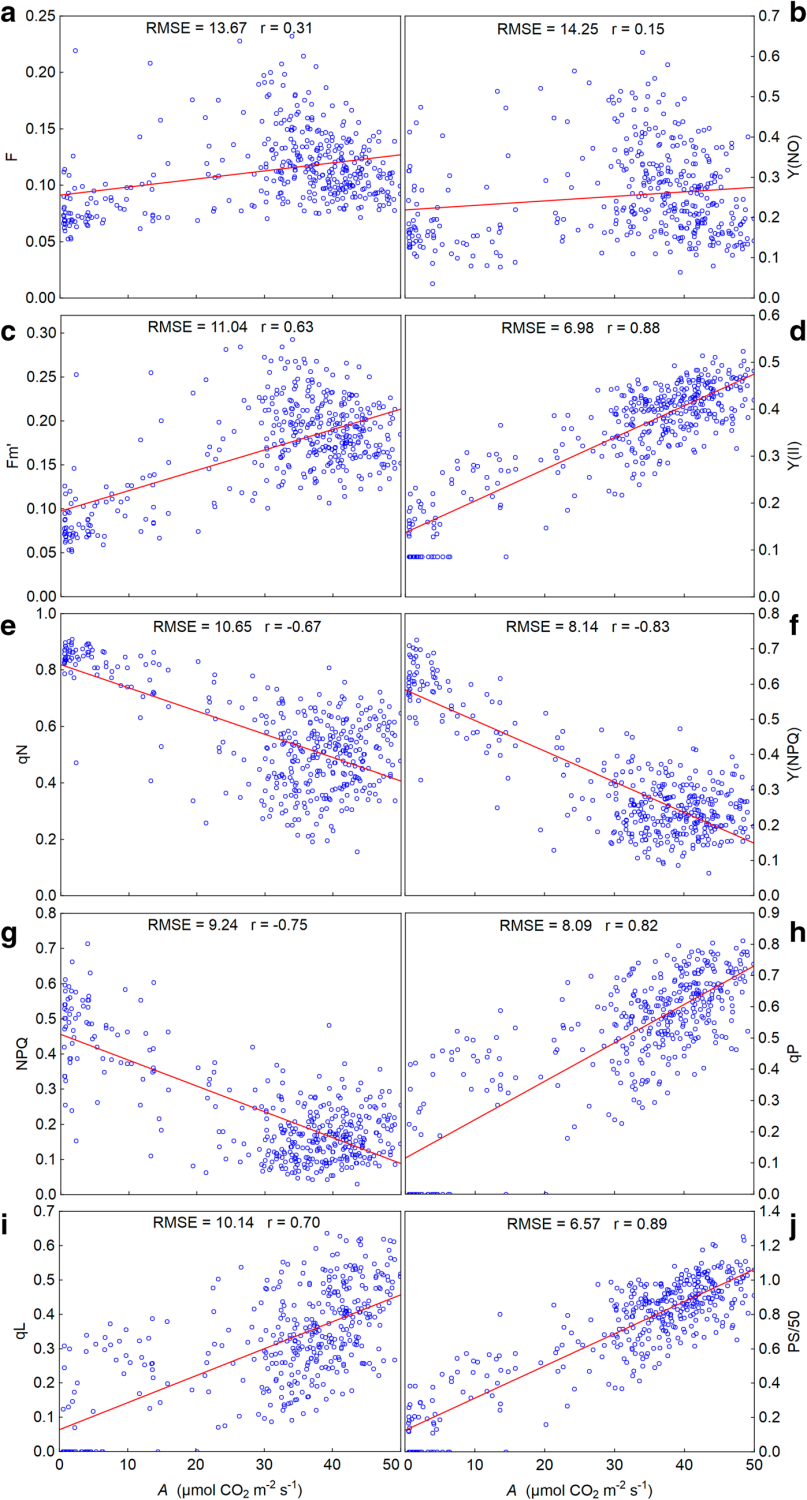
The relationship between the net CO_2_ assimilation rate (*A*) and each of the chlorophyll fluorescence parameters obtained by rapid light curves for control and drought stressed maize plants. **a** F, fluorescence yield; **b** Y(NO), quantum yield of nonregulated energy dissipation; **c** Fm’, maximum fluorescence yield on light-adapted leaf; **d** Y(II), effective PSII quantum yield; **e** qN, coefficient of non-photochemical quenching (lake model); **f** Y(NPQ), quantum yield of regulated energy dissipation; **g** NPQ, non-photochemical quenching; **h** qP, coefficient of photochemical quenching (puddle model); **i** qL, coefficient of photochemical quenching (lake model); **j** PS/50, apparent rate of photosynthesis. All chlorophyll fluorescence parameters were obtained from RLC’s in the light step at 280 µmol m^−2^ s^−1^ of actinic lighting. The data used for correlation were obtained from ten measurements over the experimental period

Since the correlation strength between the most gas exchange and chlorophyll fluorescence parameters was similar, with exception of *Ci*, we chose the rate of CO_2_ assimilation (*A*) to graph it. Thus, a scatter plot showing the strength of the correlation between *A* and the fluorescence parameters in the light step in which there was the greatest difference between stressed and control plants, i.e. at 280 µmol m^−2^ s^−1^, is shown (Fig. 3).

### Discrimination of maize genotypes responses to drought by chlorophyll fluorescence RLC’s

It is known that the changes caused by drought in the parameters of leaf gas exchange occur in parallel and interrelated to changes in chlorophyll fluorescence. Therefore, we hypothesized that these changes could be followed by the chlorophyll fluorescence parameters obtained from rapid light curves.

**Fig. 4:**
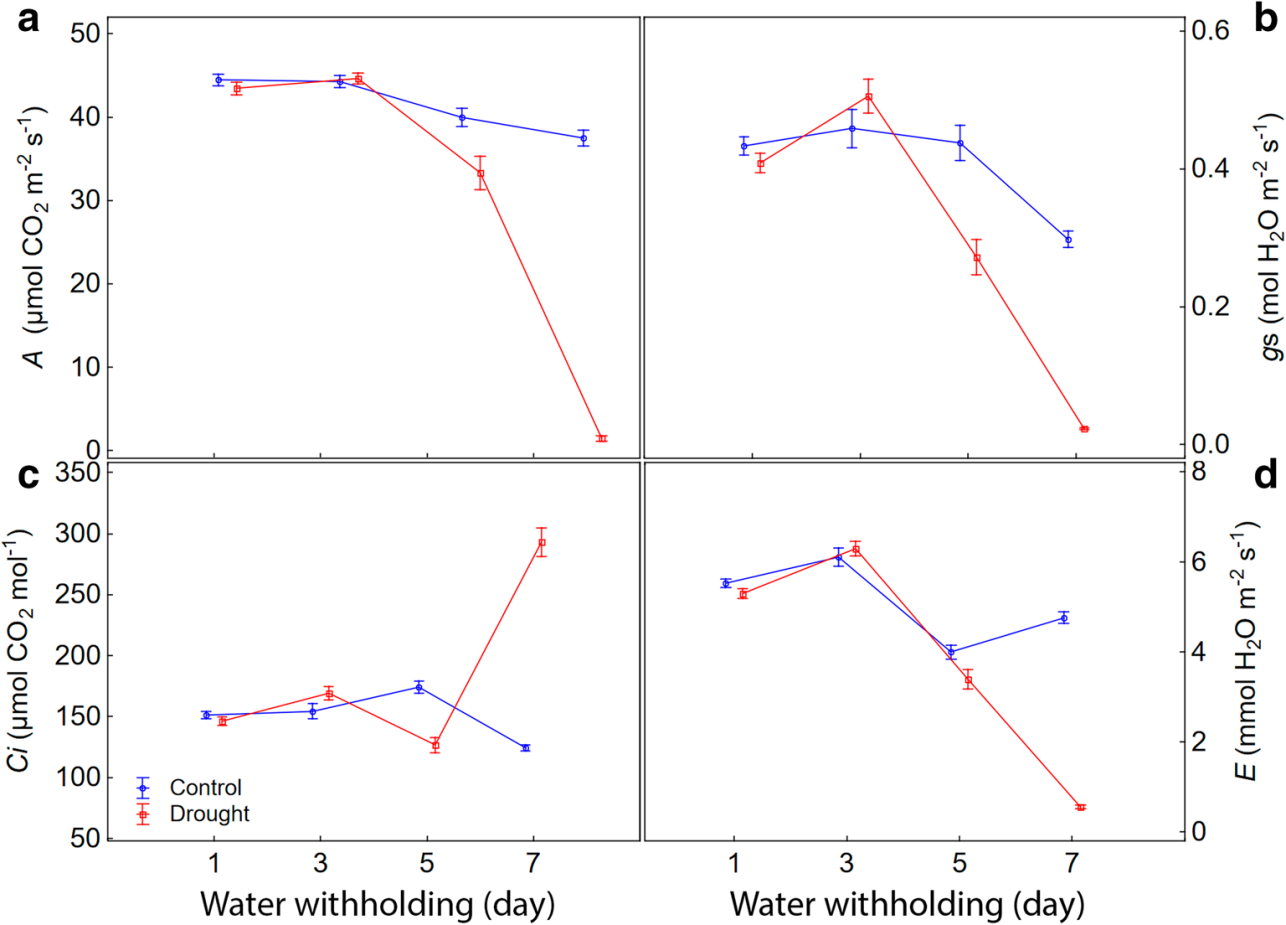
Changes over time in gas exchange parameters for control and drought stressed maize plants. **a** *A*, net CO_2_ assimilation rate; **b** *gs*, stomatal conductance to water vapor; **c** *Ci*, intercellular CO_2_ concentration; **d** *E*, transpiration rate. The values of the parameters every 2 days represent the average of 20 replicates considering all studied maize genotypes. Both groups of plants (control and drought) were with soil water content at field capacity at the start of measurement (±60 days after sowing; 1st day). From there, the watering was withheld in the drought stressed plants until the substrate reached the theoretical wilting point (−1.5 MPa) on the 7th day. *Bars* represent standard error of the mean

**Fig. 5:**
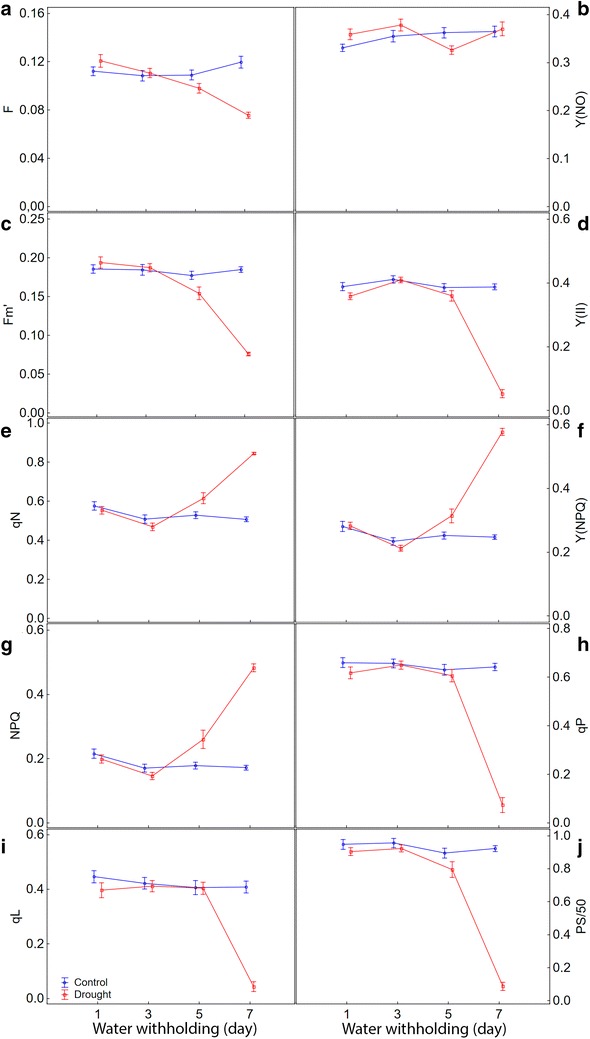
Changes over time in chlorophyll fluorescence parameters for control and drought stressed maize plants. **a** F, fluorescence yield; **b** Y(NO), quantum yield of nonregulated energy dissipation; **c** Fm’, maximum fluorescence yield on light-adapted leaf; **d** Y(II), effective PSII quantum yield; **e** qN, coefficient of non-photochemical quenching (lake model); **f** Y(NPQ), quantum yield of regulated energy dissipation; **g** NPQ, non-photochemical quenching; **h** qP, coefficient of photochemical quenching (puddle model); **i** qL, coefficient of photochemical quenching (lake model); **j** PS/50, apparent rate of photosynthesis. Data were obtained at 280 µmol m^−2^ s^−1^ of actinic lighting. The values of the parameters every 2 days represent the average of 20 replicates considering all studied maize genotypes. Both groups of plants (control and drought) were with soil water content at field capacity at the start of measurement (±60 days after sowing; 1st day). From there, the watering was withheld in the drought stressed plants until the substrate reached the theoretical wilting point (−1.5 MPa) on the 7th day. *Bars* represent standard error of the mean

The responses of the maize plants submitted to drought occurred as expected, based on the main gas exchange parameters chosen. There was a sharp drop in *A*, *gs*, and *E*, and an increase in *Ci* over 7 days after withholding (Fig. 4, Additional files 4, 5). To show that the chlorophyll fluorescence parameters follow a similar dynamic, we chose the value of each of them obtained in the light step at 280 µmol m^−2^ s^−1^ to plot over time (Fig. 5; Additional files 6, 7). Such a choice was made because it was one of the light steps less affected by induction processes. In this light step, PS/50 saturation had already been achieved on both control and stressed plants. Moreover, it was the light step in which the most parameters showed the greatest differences between stressed and non-stressed plants in the shortest time from the beginning of the RLC’s, around 60 s (Fig. 1).

**Fig. 6:**
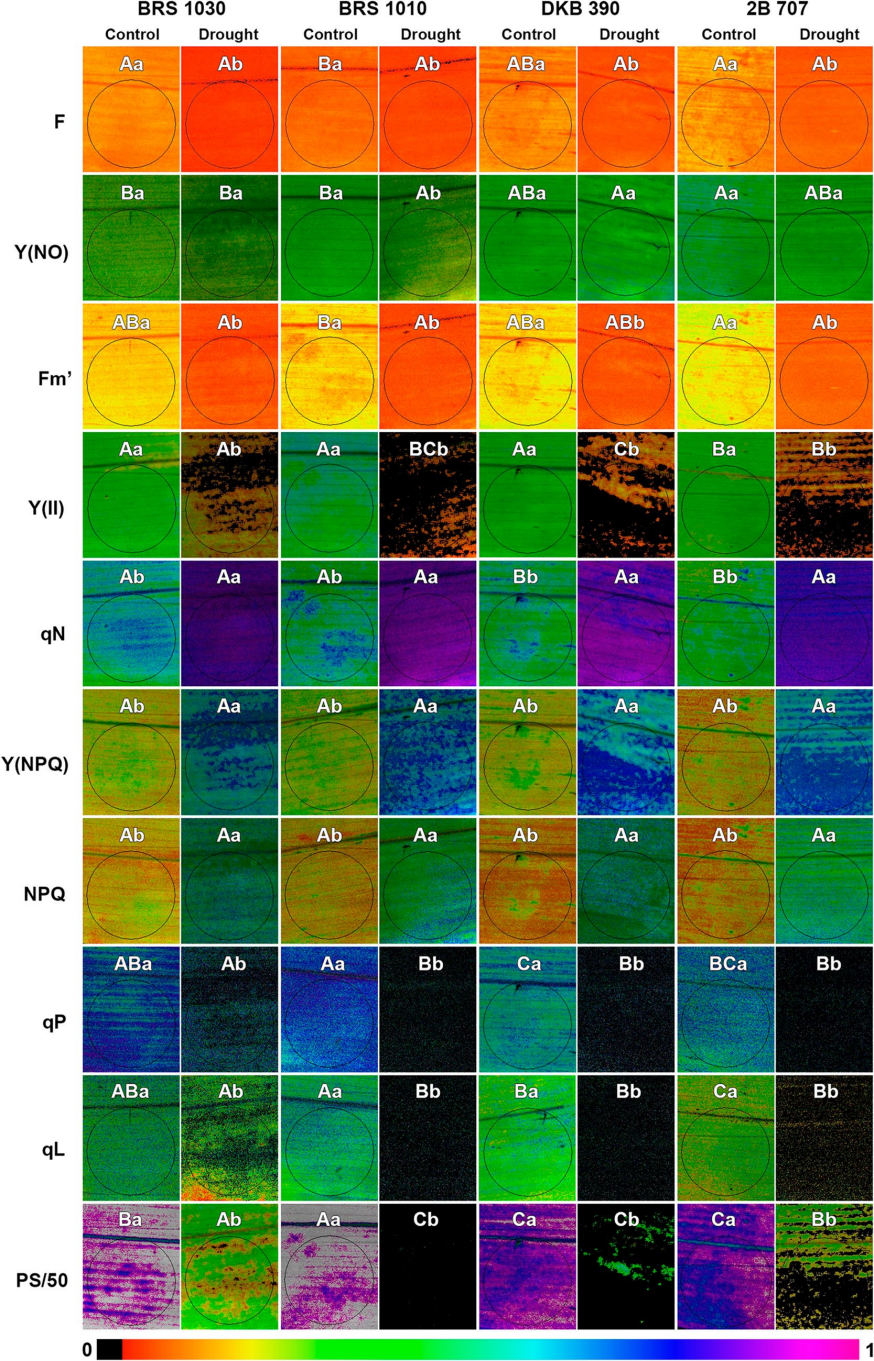
Representative images of selected areas of interest of chlorophyll fluorescence parameters for control and drought stressed maize plants. Data were obtained at 280 µmol m^−2^ s^−1^ of actinic lighting. These images were captured at the 7th day after water withholding in the stressed plants, when the water potential in the soil was around theoretical wilting point (−1.5 MPa). *Letters embedded in each figure* represent the comparison of means by Turkey’s test (p < 0.05). *The images having the same uppercase letters* indicate that the represented parameter shows no statistical differences between maize genotypes subjected to the same soil water availability. *The images having the same lowercase letters* indicate that the represented parameter shows no statistical differences between control and stressed plants to that genotype. The data in the images have been mapped to the color palette shown below

Similar to what had already occurred with the gas exchange parameters, it was observed reasonably unchanged in all chlorophyll fluorescence parameters in control plants over seven days. Also, as expected, there was a decrease in parameters related to photochemical dissipation of light energy [Y(II), qP, qL and PS/50] (Fig. 5d, h–j) consistent the extent to which the plants were entering the drought stress. The same applies to the non-normalized parameters related to fluorescence emission (F and Fm′) (Fig. 5a, c). On the other hand, the parameters related to the heat dissipation of light energy [qN, Y(NPQ) and NPQ] (Fig. 5e–g) simultaneously increased. Meanwhile, the parameter related to unregulated dissipation of light energy [Y(NO)] (Fig. 5b) virtually unchanged due to drought. At the end of the 7th day after water withholding, most parameters showed statistical differences between control and drought stressed plants which can be viewed through the images (Fig. 6; Additional files 8, 9, 10).

## Discussion

### Changes in chlorophyll fluorescence parameters over RLC’s in control and drought stressed maize plants

Clearly, there was a pattern of responses for each chlorophyll fluorescence parameter gotten along the RLC’s. This pattern repeated itself in control plants as well as in those subjected to stress by water deficit in all maize genotypes (Fig. 1; Additional file 1). It is noteworthy the initial increase followed by a reduction in F and Y(NO), both in stressed and non-stressed plants. Probably the kinetics of these parameters was influenced by the induction processes that act on the photochemical apparatus, which are affected by the procedures adopted to carry the RLC’s out. Such induction processes tend to occur, even if it takes a period of time as short as just 10 s of darkness before starting the RLC’s [43].

These results demonstrate that both stressed and control plants suffered induction effects; however, two predominantly different and competitive mechanisms were involved. Thus, the kinetic of F and Y(NO) along the RLC’s was possibly determined by the main mechanism of fluorescence quenching that each plant group have preferably used. Thus, the stressed plants, due to the limitations imposed on photosynthesis by drought, diverted a considerable part of the electrons flow to the non-photochemical processes, where induction is faster so that F and Y(NO) decreased sooner in this group of plants. On the other hand, control plants drove the electrons flow preferably for the photochemical process whose induction is slower which delayed the decline in both parameters. At the end of the RLC’s, when both processes probably were fully activated, the differences between F and Y(NO) from stressed and control plants were reduced. Even so, based on the values of these parameters, it was possible to detect statistically significant differences between stressed and control plants on the initial as well as on the end portion of the RLC’s.

The maximum fluorescence emission in the light (Fm′) is an indicative of energy dissipation by non-photochemical quenching processes, which are highly regulated in plant leaves [25]. In this study, Fm′ arbitrary values from stressed plants were lower than those of control throughout the RLC’s. However, the simple comparison of arbitrary Fm′ values of the two groups of plants would not be a good indication of the differences between them. The reason is that Fm′ is influenced by differences in chlorophyll content [32] and, at least in the beginning of the RLC’s, it also is affected by NPQ relaxation [44]. In this study, both traits were affected by drought (Figs. 1, 2). It would be more appropriate, therefore, to perform an analysis disregarding the arbitrary values of Fm′ and taking into account only the kinetics of it along the curve, which has no relation to the chlorophyll content. By using this approach, the fall in the Fm′ was much bigger and faster in plants subjected to water deficit. It is probably related to the faster start of the NPQ, which has shown much higher rates than in control plants. Similar to what occurred to F, the difference in Fm′ values between stressed and control plants was lower at the extreme ends of the RLC’s. For that reason, the intermediate portion of the RLC’s would be the most recommended part to discriminate the responses of plants to water deficit using that parameter.

The sharp reduction in the parameters related to photochemical quenching [qL, qP and Y(II)], and the concurrent and proportional increase in those related to non-photochemical quenching [qN, NPQ and Y(NPQ)], suggest that maize plants submitted to drought maintained their capacity to regulate dissipation of light energy, despite the stressful condition. This fact, along with the rapid reduction of F in this group of plants, constitutes evidence that the xanthophyll’s cycle was efficient in giving vent to excess electrons flow generated for the increase in light intensity in the chloroplasts. In fact, Saccardy et al. [44] had shown that when subjected to drought, maize plants responded by an increase in the xanthophyll’s cycle pool size as well as the in the proportion of de-epoxidized (antheraxanthin and zeaxanthin) to epoxidized (violoxanthin) xanthophylls.

Higher pressure on the photochemical apparatus, represented by an increase in light intensity along the RLC’s, had only a limited effect on the enlargement of the difference in Y(II) or PS/50 between control and drought plants. PS/50 reached saturation around 280 µmol m^2^ s^−1^ in control plants, while in those stressed saturation occurred in a lower light intensity, around 185 µmol m^2^ s^−1^. The increase in the difference between these two groups of plants could, in this case, only occur if Y(II) and, consequently, PS/50 fell to zero in stressed plants. This possibility would depend mainly on the increase in stress intensity and on the genotype sensitivity, but not by an increase in light intensity. In fact, after reaching saturation, an increase in light intensity would have an opposite effect, reducing the difference between these groups of plants, as a consequence of the drop in both Y(II) and PS/50, mainly in control plants. Based on this found, there is no need to move beyond 280 µmol m^2^ s^−1^ for the purpose of discriminating the maize genotypes responses to drought by using these two parameters. On the other hand, smaller light intensity values do not express the maximum potential difference that can exist between the two groups of plants.

### RLC’s chlorophyll fluorescence parameters enabling discrimination of maize genotypes responses to drought

Based on the results of this study, we found that we must be careful in interpretation and using parameters obtained by RLC’s to discriminate plant responses to drought. The use of such parameters for that purpose only because they have shown statistically significant correlation with those of gas exchange (Table 1; Fig. 3), or due to the fact they have presented differences between control and stressed plants (Figs. 1, 5, 6), can lead to errors. It is important that their use for this purpose be supported by the knowledge of what each of them actually represents, and to what trait in the plant it is linked. In addition, one should take into account that secondary factors, which are associated with drought, may interfere with chlorophyll fluorescence parameters obtained by RLC’s.

F, Fm′ and Y(NO) well illustrate this point. The low correlation coefficients for F and Y(NO), both related to light energy dissipation in a non-regulated manner [16, 26], for instance, indicate that this process does not maintain a strong link with the leaf gas exchange. Furthermore, the analysis of the results from non-normalized parameters, such as F and Fm′, becomes complicated. They were affected by the loss of chlorophyll from the leaves throughout the drought period (Fig. 2), with a probable and consequent reduction in the amount of absorbed light [19]. It must also be considered a remarkable effect of induction over these parameters, observed throughout the actinic lighting steps from RLC’s. As a result, depending on the level of light used for comparison, stressed plants can have a smaller or larger values of F and Y(NO) in comparison to controls. This makes these parameters somewhat ambiguous to discriminate plant responses to drought.

The parameters related to the photochemical [Y(II), qP and qL] and non-photochemical [qN, NPQ, and Y(NPQ)] quenching showed, differently of the parameters linked to fluorescence emission, strong correlation with those derived from gas exchange. According to the theory of partitioning of absorbed light energy among photochemistry, fluorescence and heat, Y(II) measures the proportion of the light energy absorbed by PSII which is used in photochemistry [13, 14, 17, 24, 25]. As such, it can give a measure of the rate of linear electron transport from PSII to PSI. Consequently, there is a strong linear relationship between this parameter and quantum efficiency of carbon assimilation [45].

By limiting photochemistry, as occurred in drought maize plants, Y(II) would decrease in direct proportion of CO_2_ assimilation, whereas heat dissipation would increase in the inverse proportion. For the first premise to be true, there must be no change in fluorescence yield [Y(NO)], which was actually confirmed (Fig. 5b). In addition, other electron drains [45–47] cannot be functional. Indeed, there is no evidence in some studies that other processes such as photorespiration or Mehler reaction may be involved in fluorescence extinction in maize plants under drought stress [44, 46]. And, even though if there may have been other electrons drains, as suggested by others studies on maize under stress [47], including drought [48, 49], in this study they were not strong enough to prevent the correlation among gas exchange and chlorophyll fluorescence parameters.

It was found that any of these normalized photochemical and non-photochemical parameters obtained by RLC’s were able to discriminate maize plant responses to drought. Therefore, any of them can be used for such purpose. This result is independent on whether or not the measurement was performed using the actual values of chlorophyll fluorescence parameters. However, not all light steps can be recommended for this because the correlation coefficients from all parameters varied along the RLC’s, depending on the intensity of actinic light. They were lower in the initial steps of RLC’s, but increased along with actinic light, reaching the highest values as soon as the light reached saturation, around 280 µmol m^2^ s^−1^. From there, any increase in light intensity virtually had no effect on correlation coefficients.

It is known there is a spatial heterogeneity in chlorophyll fluorescence parameters along the leaf blade of plants, regardless of the previous conditions under which they were submitted [16, 25, 36, 50, 51]. This phenomenon was also observed along the maize leaf blade (Fig. 6), which was more pronounced in certain parameters, such as Y(II) (Additional file 9) and PS/50 (Additional file 10). Taking this into account, in addition to the unfeasibility of measuring the whole leaf section, we were careful to choose the area of interest (AOI) of the leaves to be evaluated (see “Methods”). In this same AOI, dozens of data obtained from several replicates were used. Therefore, irrespective of heterogeneity, we showed there was a strong correlation between parameters of gas exchanges with those of chlorophyll fluorescence. Finally, we emphasize that the central point of the manuscript was to show that it is possible to discriminate responses of maize genotypes to drought based on RLC’s chlorophyll fluorescence measurements.

## Conclusions

Almost all chlorophyll fluorescence parameters generated by RLC’s showed differences between control and drought maize genotypes. However, not all parameters and nor all light steps could be used for correct discrimination to drought. Most parameters were affected by induction processes, relaxation rate, and/or differences in chlorophyll content; while they all were influenced by actinic light intensity on each light step of light curve. Only normalized parameters relative to photochemical and non-photochemical quenching, measured in the light step in which the linear electron flow reached saturation, were indicated for this. Such measurements were as fast, efficient and reliable as the standard technique of gas exchange in order to discriminate the responses of maize genotypes to stress by water deficit. These findings are important as theses parameters can be used for discrimination of plants subjected to drought both in controlled and field conditions. This may represent an advance in the current trend of large-scale plant phenotyping by imaging systems.

## Methods

### Plant material and growing conditions

The experiments were carried out in a greenhouse at National Agroenergy Research Center (https://www.embrapa.br/en/agroenergia), in Brasilia, Brazil (S—15.732°, W—47.900°) from November 2015 to February 2016. Three weather variables (light intensity, temperature, and humidity) fluctuated accordingly to environmental conditions. Only water supply to the plants and soil moisture content were controlled. Four maize genotypes showing contrasting responses to drought based only on grain yield were used: susceptible (BRS 1010), intermediate (BRS 1030) and tolerant (DKB 390 and 2B 707). They were chosen based on previous results from experiments carried out at National Maize and Sorghum Research Center (https://www.embrapa.br/en/milho-e-sorgo) under field conditions. The seeds were sown in plastic pots (20 kg), filled with typical soil used for maize cultivation in the region (dystrophic Red Latosol according to Brazilian Soil Classification), limed and fertilized based on results from a physical–chemical analysis. After germination, two plants were held per pot, on a daily replenishment of water at field capacity (100% of the available water), based on the weight.

### Drought stress and assessed leaf

Stress treatment by drought started about 60 days after sowing, at the pre-flowering stage, when all the plants reached the vegetative growth stage V16 (16 leaf collars emitted). A group of five plants from each genotype was subjected to drought stress by withholding water until the water potential decreased to about −1.5 MPa. They were kept in these conditions for 12 consecutive days, while another group of five plants of the same genotype remained fully irrigated (control). All chlorophyll fluorescence, gas exchange and chlorophyll content index measurements were performed on the adaxial side of a healthy and fully expanded leaf 16, in a previously marked area of approximately 40 cm^2^ (Additional file 11). This area was located in the middle third of the leaf, at a distance of 25 cm from the apex. It had been chosen in pilot experiments because: (1) it presented highest photosynthetic rates based on gas exchange measurements; (2) under control conditions, it presented lower variation in photosynthetic rates among individuals of the same genotype and even among different genotypes, as can be seen in the data; (3) It is located at a certain distance from the apex, the region most affected by drought stress. Such characteristics are important for studying long-term stresses in which the same attached leaf is evaluated over time.

### Chlorophyll fluorescence parameters

Plants submitted to drought stress were assessed by the chlorophyll fluorescence technique (Saturation Pulse Method). For this, chlorophyll a fluorescence was measured using an imaging fluorimeter Walz model IMAGING-PAM Maxi version (Heinz Walz GmbH, Effeltrich, Bayern, Germany) driven by the ImaginWin version 2.40b software. The measuring head consisting of LED-Array Illumination Unit IMAG-MAX/L and a CCD camera IMAG-MAX/K4 was mounted on IMAG-MAX/GS stand. The setup was set on a tripod to make easier the adjustment of the measuring head height for matching the height of the plant to be imaged (Additional file 12). The following settings were used: measuring light = 1; saturation pulse = 10 (2800 µmol m^−2^ s^−1^); gain = 1; dumping = 2; red gain = 25; red intensity = 4; NIR intensity = 7; Fm factor = 1.055; F factor = 0.999. For measuring the chlorophyll fluorescence parameters, upward light curves were used. The measurements were performed on individual attached leaves placed under the measuring head and kept in the dark for about 10 s. After that, a light curve with increasing actinic light steps (0, 20, 55, 110, 185, 280, 335, 395, 460) was initiated, using blue light provided by LEDs in 10 s intervals, at the end of which it was applied a saturation pulse. During RLC’s running, a black fabric was used to cover the measuring head in order to avoid external light input at the sample stage. The chlorophyll fluorescence measurements were performed between 9:00 and 12:00 a.m. in a leaf circular area (approximately 12 cm^2^). This area of interest (AOI) represents the center of the previously marked area in which all measurements were performed, as described in the previous section. It is a circular area that encompasses just half width of the leaf to avoid midrib. The initial values of F and Fm′ obtained in the RLC’s at the light step zero were considered to be Fo and Fm just in order to calculate parameters related to the photochemical and non-photochemical quenching. However, they do not represent the actual values of those parameters, which are obtained when the PSII reaction center is completely oxidized. Also, for the calculation of parameters that depend on it, such as Y(II), the F values were considered as Fs. From the measured parameters, all derived parameters were calculated according to the equations described in the equipment manual, from the ImaginWin version 2.40b software. The Y(II) was determined using the equation: Y(II) = (Fm′ − Fs)/Fm′, as originally described by Genty et al. [45]. Other equations were basically the same described in Van Kooten and Snel [27] and Maxwell and Johnson [28] as follow: qP = (Fm′ − Fs)/(Fm′ − Fo′), qN = 1 − (Fm′ − Fo′)/(Fm − Fo), NPQ = (Fm − Fm′)/Fm′. The qL, Y(NPQ) and Y(NO) were determined according to the equations described by Kramer et al. [26]: qL = qP × Fo′/Fs, Y(NPQ) = 1 − Y(II) − 1/(NPQ + 1 + qL(Fm/Fo − 1)), Y(NO) = 1/(NPQ + 1 + qL(Fm/Fo − 1)). Instead of electron transport rate (ETR), the ImaginWin software generates images of a similar parameter denominated PS which is calculated according to the equation: [PS = 0.5 × Y(II) × PAR × Abs.]. In order to display images of this parameter on a false color scale ranging from 0 to 1, the PS value was divided by a number, which correspond to the expected limit of maximal PS. For this study, it was used the standard setting which is 50 and the resulting parameter is called PS/50. This means that the pixel value 1 is reached when PS/50 = 1. The amount of light absorption determined by routine absorptivity was used for the calculation of PS/50. The absorptivity (Abs.) value represents a measure of the fraction of the incident red light which is absorbed by the leaf. It was calculated by the following equation: Abs. = 1 − R/NIR. The values of calculated and measured chlorophyll fluorescence parameters represent the mean values of all pixels within an AOI.

### Gas exchange parameters

An LI-COR 6400XT (LI-COR, Lincoln, NE, USA) infra-red gas analyzer, equipped with a size measuring head with 2 × 3 cm and a lighting system artificial LED model 6400-02B was used for measuring of gas exchange. The equipment was configured to maintain the relative humidity within the measuring chamber between 50 and 60% at 30 °C, light intensity in 2000 μmol m^−2^ s^−1^ and flow rate at 500 μmol s^−1^ while collecting data. CO_2_ concentration was maintained at 400 ppm using a CO_2_ mixer model 6400-01 from cylinder (12 g) CO_2_. Each gas exchange measurement in each maize leaf was carried out before chlorophyll fluorescence measurement. By using equations described in the LI-COR 6400XT user manual, the following parameters were obtained by means of software OPEN version 6.3: A = net CO_2_ assimilation rate (µmol CO_2_ m^−2^ s^−1^), *gs* = stomatal conductance to water vapor (mol H_2_O m^−2^ s^−1^), *E* = transpiration rate (mmol H_2_O m^−2^ s^−1^) and *C*_*i*_ = intercellular CO_2_ concentration (µmol CO_2_ mol air^−1^).

### Chlorophyll content index

Chlorophyll content index (CCI) was measured using a chlorophyll meter Opti-science model CCM-200 Plus (Opti-Sciences Inc., Hudson, NH, USA). In the selected area of the leaf (see Additional file 10) 5 measures were performed. The average of these measures represented the CCI of the leaf.

### Experimental design and statistical analysis

A completely randomized design was used to assign two treatments (control × drought) in four maize genotypes (BRS 1030, BRS1010, DKB 390 and 2B 707) and five replicates. Data were analyzed using a one-way analysis of variance (ANOVA). When the treatments or their interactions were significant, the means were compared by Tukey’s test (p ≤ 0.05). Pearson’s product moment correlation coefficients were used to estimate relationships between gas exchange and chlorophyll fluorescence parameters. All the statistical and correlations analyses were performed by using the statistical program STATISTICA version 12 (www.statsift.com, Tulsa, OK, USA).

## Additional files

**Additional file 1.** Chlorophyll fluorescence parameters obtained by RLC’s applied to the leaves of the four different maize genotypes grown under control or drought conditions as a function of PAR. All maize plants were grown with soil water content at field capacity. At the V16 stage, a group of plants of each genotype was subjected to water withholding until reach the theoretical wilting point (drought) and remaining for 12 days while another group was kept under field capacity (control). The value of each parameter in each light step represents the average of four measurements over the period of stress for each maize genotype studied under control or drought stress. Bars represent standard error of the mean.

**Additional file 2.** (A) Resume of variance analyses from chlorophyll fluorescence parameters obtained by RLC’s in leaves of four the different maize genotypes grown under control or drought stress as a function of PAR. (B) Comparison of means by Tukey’s test (p < 0.05) from chlorophyll fluorescence parameters in the same RLC’s light step in leaves of maize genotypes grown under control or drought conditions. (C) Multi comparison of means by Tukey’s test (p < 0.05) from chlorophyll fluorescence parameters over the RLC’s light steps obtained in the leaves of maize genotypes continuously grown under soil water available at field capacity (control). (D) Multi comparison of means by Tukey’s test (p < 0.05) from chlorophyll fluorescence parameters over the RLC’s light steps obtained in leaves of maize genotypes in which the plants were subjected to water withholding (drought).

**Additional file 3.** Changes over time in leaf chlorophyll content index for control and drought stressed maize genotypes under study. The values of the parameters represent the average of five replicates for each studied maize genotypes. Both groups of plants (control and drought) were held with soil water content at field capacity at the start of measurement (±60 days after sowing; 1st day). From there, the watering was withheld in the drought stressed plants until the substrate reached the theoretical wilting point (−1.5 MPa) on the 7th day. Bars represent standard error of the mean.

**Additional file 4.** Changes over time in leaf gas exchange parameters for control and drought stressed study maize genotypes under study. The values of the parameters represent the average of five replicates for each studied maize genotype. Both groups of plants (control and drought) were with soil water content at field capacity at the start of measurement (day 1). From there, the watering was withheld in the drought stressed plants until the substrate reached the theoretical wilting point (−1.5 MPa) on the 7th day. Bars represent standard error of the mean.

**Additional file 5.** (A) Resume of variance analyses from gas exchange parameters obtained in leaves of four different maize genotypes grown under control or drought stress as a function of time. (B) Comparison of means by Tukey’s test (p < 0.05) from gas exchange parameters in leaves of maize genotypes grown under control or drought conditions. (C) Multi comparison of means by Tukey’s test (p < 0.05) from gas exchange parameters over time in the leaves of maize genotypes continuously grown under soil water available at field capacity (control). (D) Multi comparison of means by Tukey’s test (p < 0.05) from gas exchange parameters over time in the leaves of maize genotypes in which the plants were subjected to water withholding (drought). Both groups of plants (control and drought) were with soil water content at field capacity at the start of measurement (day 1). From there, the watering was withheld in the drought stressed plants until the substrate reached the theoretical wilting point (−1.5 MPa) on the 7th day.

**Additional file 6.** Changes over time in leaf chlorophyll fluorescence parameters obtained by the RLC’s for control and drought stressed maize genotypes under study. The values of the parameters represent the average of five replicates for each studied maize genotype. Both groups of plants (control and drought) were with soil water content at field capacity at the start of measurement (day 1). From there, the watering was withheld in the drought stressed plants until the substrate reached the theoretical wilting point (−1.5 MPa) on the 7th day. Bars represent standard error of the mean.

**Additional file 7.** (A) Resume of variance analyses from chlorophyll fluorescence parameters obtained in leaves of four different maize genotypes grown under control or drought stress as a function of time. (B) Comparison of means by Tukey’s test (p < 0.05) from chlorophyll fluorescence parameters in leaves of maize genotypes grown under control or drought conditions. (C) Multi comparison of means by Tukey’s test (p < 0.05) from chlorophyll fluorescence parameters over time in the leaves of maize genotypes continuously grown under soil water available at field capacity (control). (D) Multi comparison of means by Tukey’s test (p < 0.05) from chlorophyll fluorescence parameters over time in the leaves of maize genotypes in which the plants were subjected to water withholding (drought). Only the data obtained at 280 µmol m^−2^ s^−1^ of actinic illumination were used. Both groups of plants (control and drought) were with soil water content at field capacity at the start of measurement (day 1). From there, the watering was withheld in the drought stressed plants until the substrate reached the theoretical wilting point (−1.5 MPa) on the 7th day.

**Additional file 8.** (A) Resume of variance analyses from chlorophyll fluorescence parameters obtained by RLC’s applied to the leaves of different maize plants grown under control or drought conditions obtained at the 7th day after water withholding. Only the data obtained at 280 µmol m^−2^ s^−1^ of actinic illumination were used. (B) Comparison of means by Tukey’s test (p < 0.05) from chlorophyll fluorescence parameters in the leaves of the same maize genotype grown under control or drought conditions. (C) Comparison of means by Tukey’s test (p < 0.05) from chlorophyll fluorescence parameters in the leaves of different maize genotypes continuously grown under soil water available at field capacity (control). (D) Comparison of means by Tukey’s test (p < 0.05) from chlorophyll fluorescence parameters in the leaves of different maize genotypes in which the plants were subjected to water withholding (drought).

**Additional file 9.** Representative images of whole leaf area measured of effective quantum yield of the photosystem II for control and drought stressed maize plants leaves. These images correspond to the measured area of the maize leaves, from which the selected areas of interest shown in Fig. 6 were taken. The data in the images have been mapped to the color palette shown below.

**Additional file 10.** Representative images of whole leaf area measured of apparent rate of photosynthesis for control and drought stressed maize plants leaves. These images correspond to the measured area of the maize leaves, from which the selected areas of interest shown in Fig. 6 were taken. The data in the images have been mapped to the color palette shown below.

**Additional file 11.** Illustrative image of maize leaves areas selected for the different measurements. The space between the two dark bars represents the area of 40 cm^2^ chosen for all measurements (see “Methods”). The sites in which the measurements of chlorophyll content index, chlorophyll fluorescence and gas exchange were performed are indicated by smaller open black circles, larger open blue circle and white rectangle circle inside the blue circle, respectively.

**Additional file 12.** Digital image showing a maize plant being evaluated by IMAGING-PAM. For the measurement, an attached leaf was placed in the sample stage inside the measuring head which was closed and covered with a black fabric to prevent external light.

## Abbreviations

Fo, Fo′: minimum fluorescence yield on dark- and light-adapted leaf, respectively
F: fluorescence yield (not necessarily in the steady-state) before application a saturate pulse
Fs: fluorescence yield on light-adapted leaf (steady-state) before application a saturate pulse
Fm, Fm′: maximum fluorescence yield on dark- and light-adapted leaf, respectively
Fv/Fm: maximum PSII quantum yield
Y(II): effective PSII quantum yield
Y(NPQ): quantum yield of regulated energy dissipation
Y(NO): quantum yield of nonregulated energy dissipation
Abs.: measuring of absorption light by the leaf
NPQ: non-photochemical quenching
qN: coefficient of non-photochemical quenching (lake model)
qP: coefficient of photochemical quenching (puddle model)
qL: coefficient of photochemical quenching (lake model)
RLC: rapid light curve
PSI, PSII: photosystem I and photosystem II, respectively
ETR: electron transport rate
PS/50: apparent rate of photosynthesis
*A*: net CO_2_ assimilation rate
*gs*: stomatal conductance to water vapor
*E*: transpiration rate
*C*_*i*_: intercellular CO_2_ concentration
CCI: chlorophyll content index
