# Supplementary figures and images for: A procedure for maize genotypes discrimination to drought by chlorophyll fluorescence imaging rapid light curves
Source: Plant Methods. 2017 Jul 26;13:61. doi: 10.1186/s13007-017-0209-z (PMC5530575; doi:10.1186/s13007-017-0209-z)

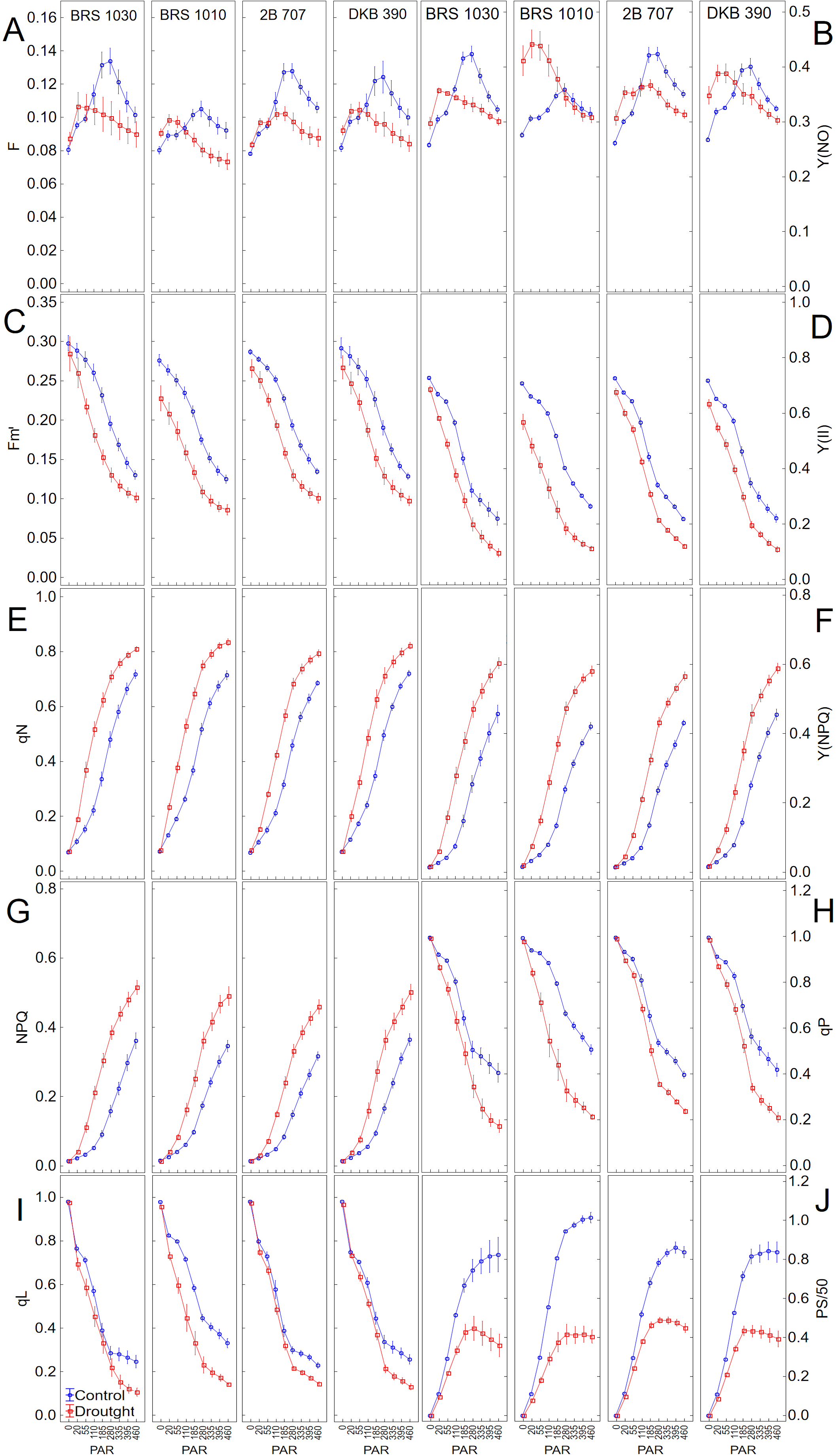

Supplement: Supplementary file 1 — Additional file 1. Chlorophyll fluorescence parameters obtained by RLC’s applied to the leaves of the four different maize genotypes grown under control or drought conditions as a function of PAR. All maize plants were grown with soil water content at field capacity. At the V16 stage, a group of plants of each genotype was subjected to water withholding until reach the theoretical wilting point (drought) and remaining for 12 days while another group was kept under field capacity (control). The value of each parameter in each light step represents the average of four measurements over the period of stress for each maize genotype studied under control or drought stress. Bars represent standard error of the mean. [file 13007_2017_209_MOESM1_ESM.tif]

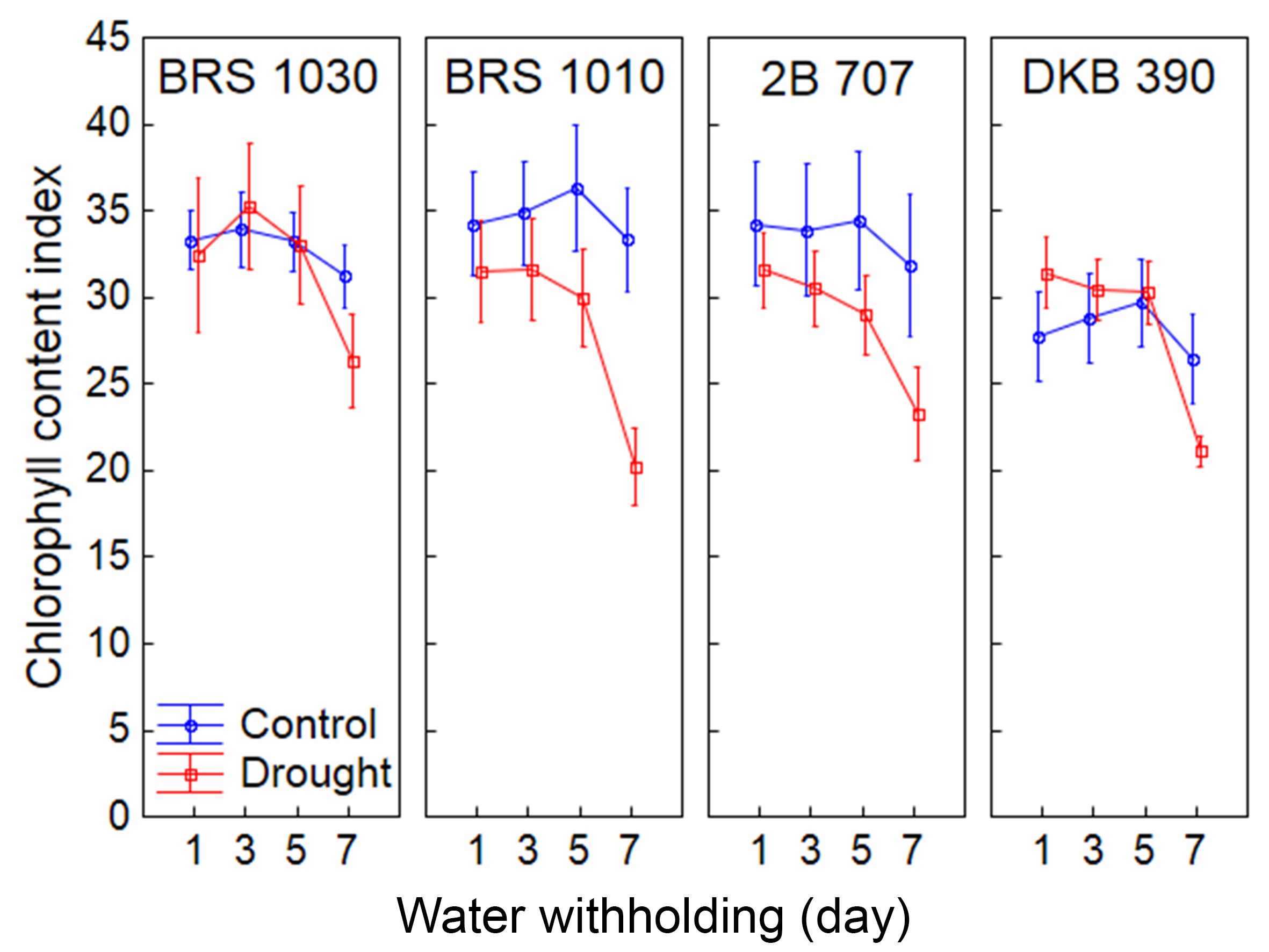

Supplement: Supplementary file 3 — Additional file 3. Changes over time in leaf chlorophyll content index for control and drought stressed maize genotypes under study. The values of the parameters represent the average of five replicates for each studied maize genotypes. Both groups of plants (control and drought) were held with soil water content at field capacity at the start of measurement (±60 days after sowing; 1st day). From there, the watering was withheld in the drought stressed plants until the substrate reached the theoretical wilting point (−1.5 MPa) on the 7th day. Bars represent standard error of the mean. [file 13007_2017_209_MOESM3_ESM.tif]

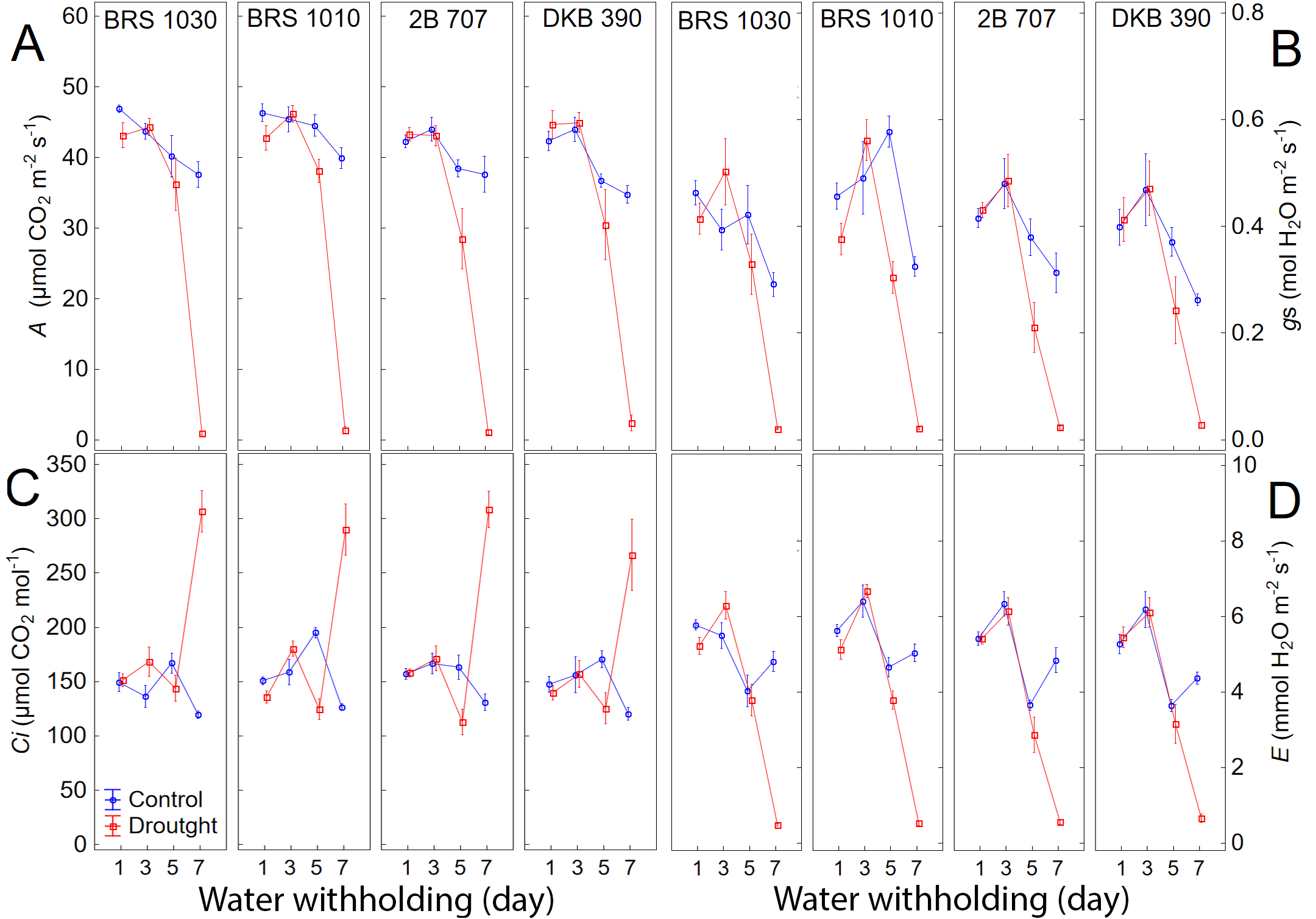

Supplement: Supplementary file 4 — Additional file 4. Changes over time in leaf gas exchange parameters for control and drought stressed study maize genotypes under study. The values of the parameters represent the average of five replicates for each studied maize genotype. Both groups of plants (control and drought) were with soil water content at field capacity at the start of measurement (day 1). From there, the watering was withheld in the drought stressed plants until the substrate reached the theoretical wilting point (−1.5 MPa) on the 7th day. Bars represent standard error of the mean. [file 13007_2017_209_MOESM4_ESM.tif]

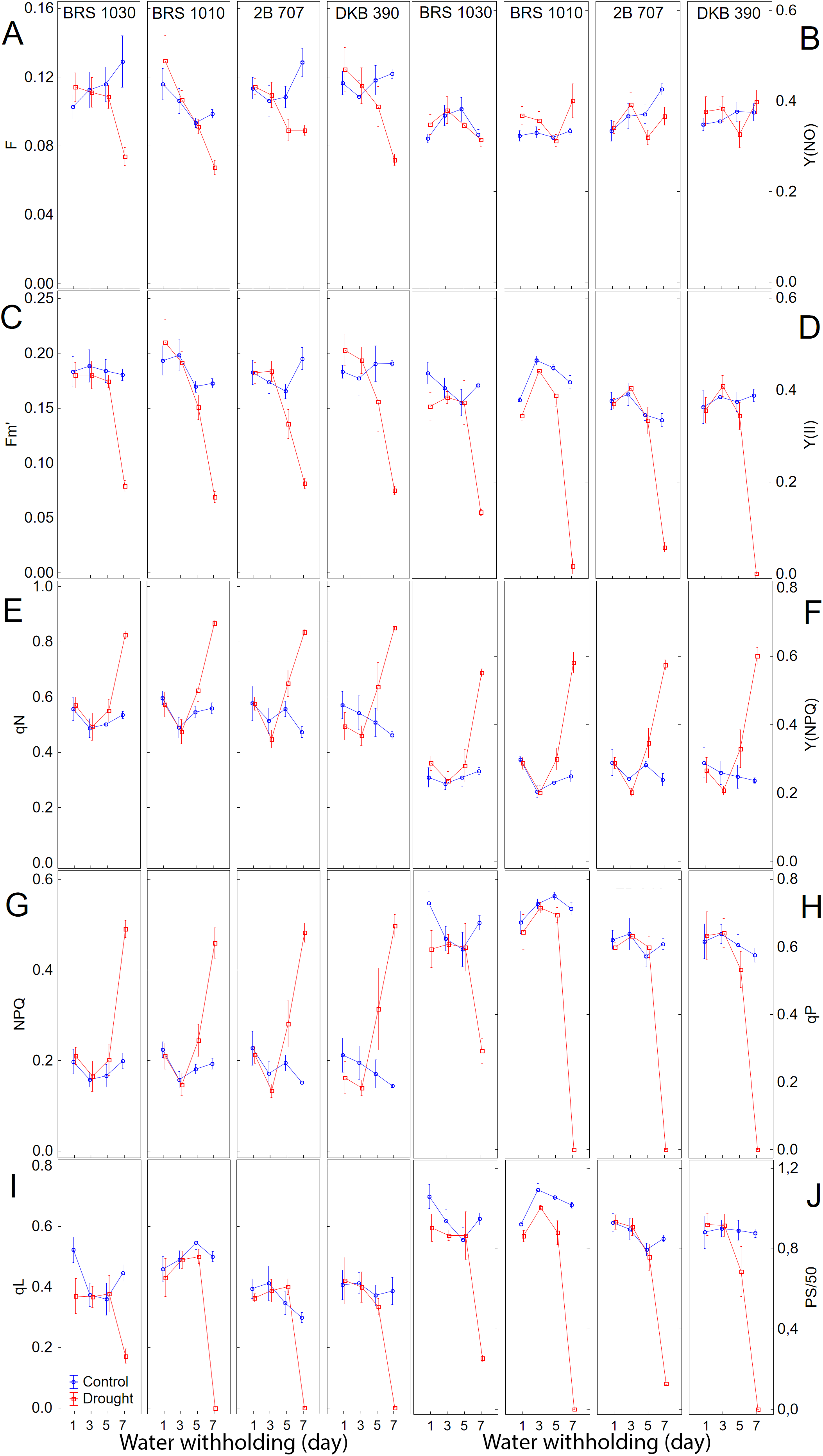

Supplement: Supplementary file 6 — Additional file 6. Changes over time in leaf chlorophyll fluorescence parameters obtained by the RLC’s for control and drought stressed maize genotypes under study. The values of the parameters represent the average of five replicates for each studied maize genotype. Both groups of plants (control and drought) were with soil water content at field capacity at the start of measurement (day 1). From there, the watering was withheld in the drought stressed plants until the substrate reached the theoretical wilting point (−1.5 MPa) on the 7th day. Bars represent standard error of the mean. [file 13007_2017_209_MOESM6_ESM.tif]

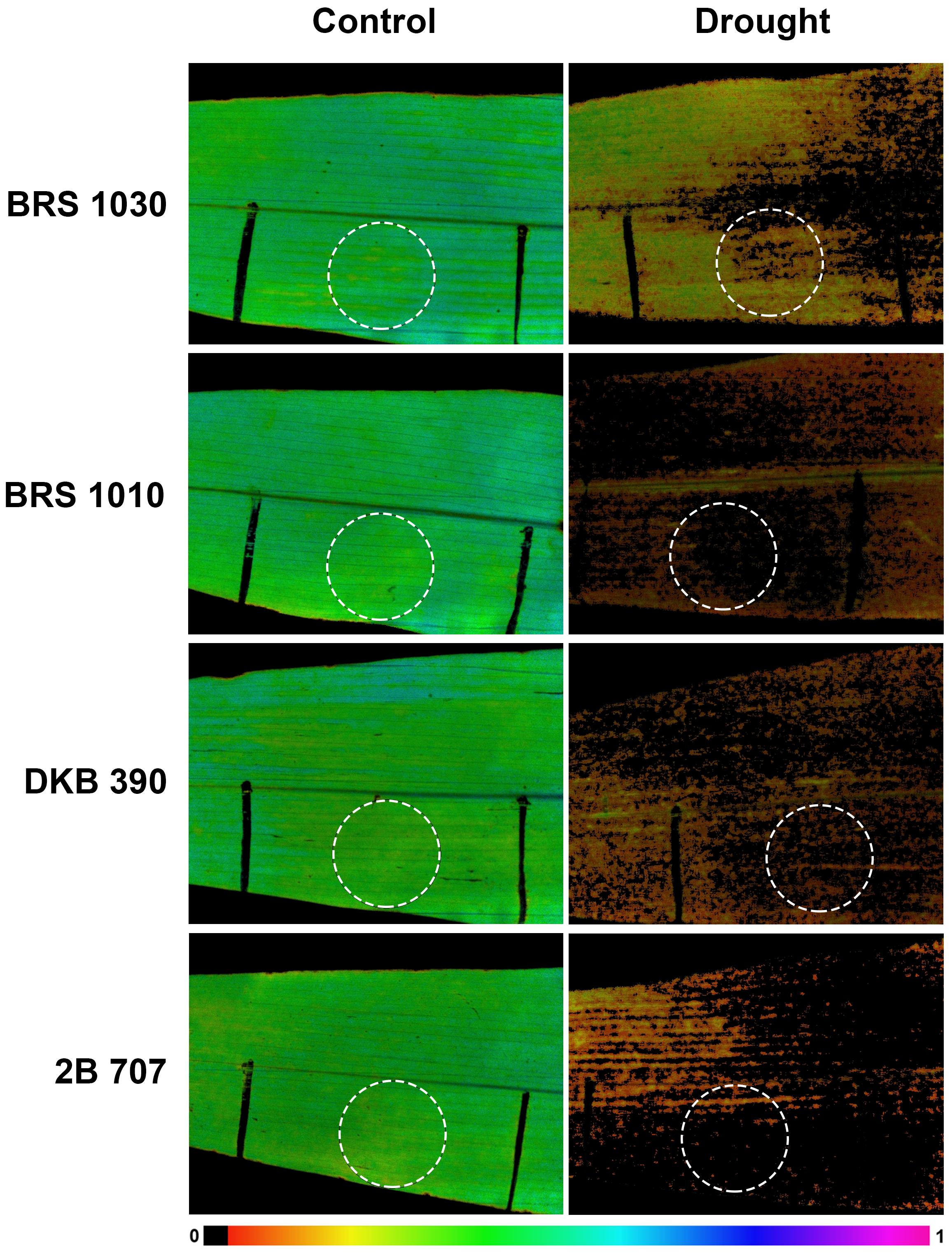

Supplement: Supplementary file 9 — Additional file 9. Representative images of whole leaf area measured of effective quantum yield of the photosystem II for control and drought stressed maize plants leaves. These images correspond to the measured area of the maize leaves, from which the selected areas of interest shown in Fig. 6 were taken. The data in the images have been mapped to the color palette shown below. [file 13007_2017_209_MOESM9_ESM.tif]

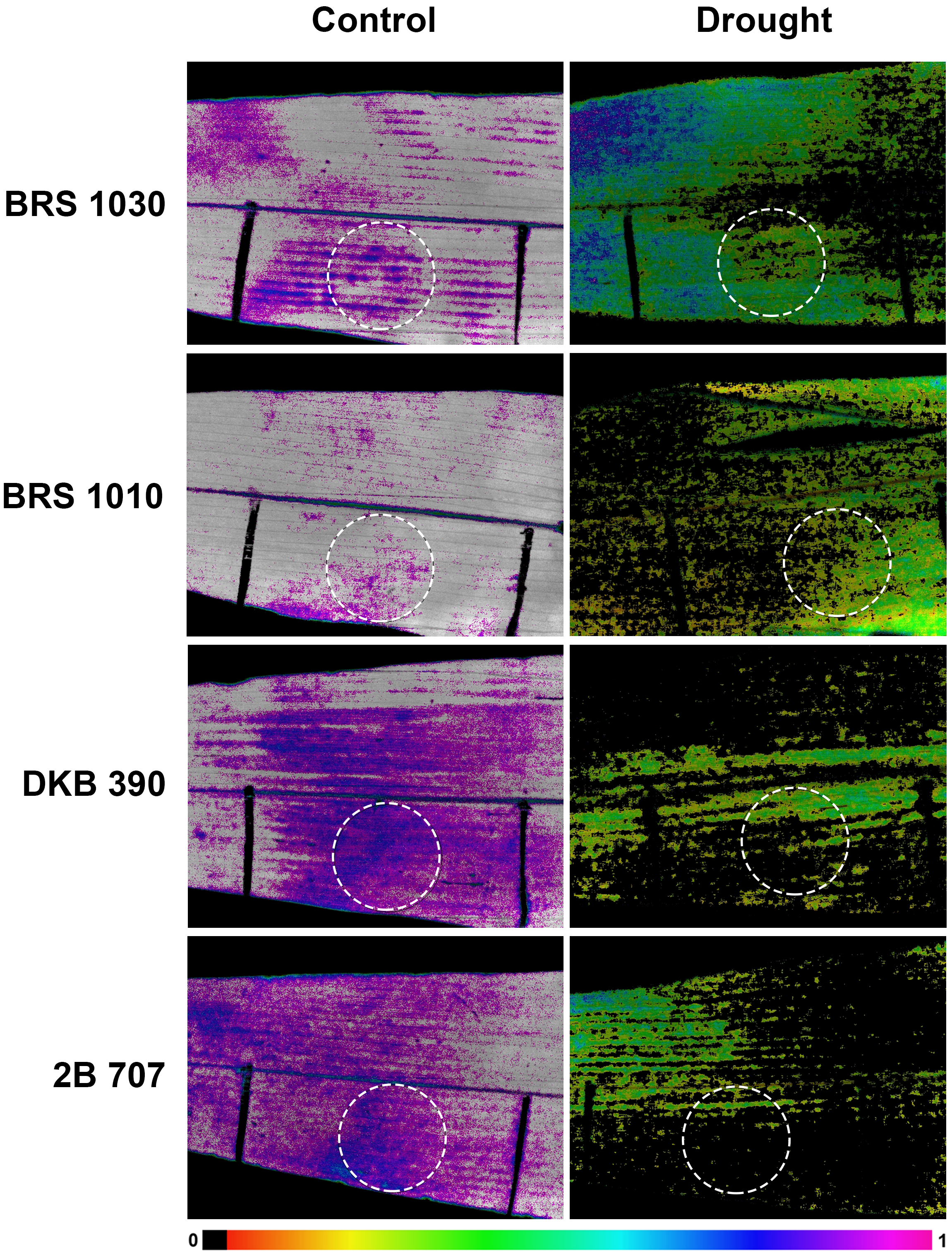

Supplement: Supplementary file 10 — Additional file 10. Representative images of whole leaf area measured of apparent rate of photosynthesis for control and drought stressed maize plants leaves. These images correspond to the measured area of the maize leaves, from which the selected areas of interest shown in Fig. 6 were taken. The data in the images have been mapped to the color palette shown below. [file 13007_2017_209_MOESM10_ESM.tif]

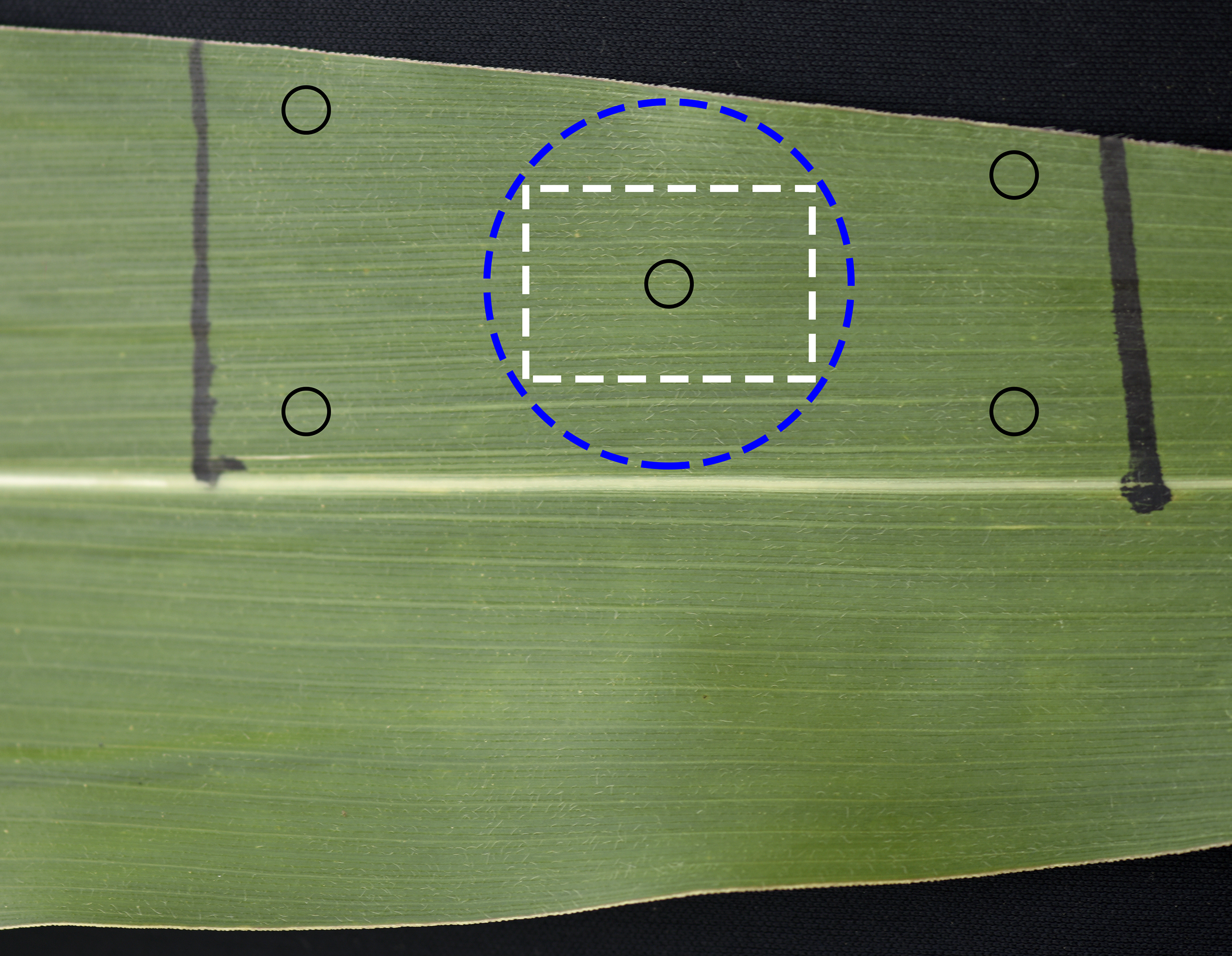

Supplement: Supplementary file 11 — Additional file 11. Illustrative image of maize leaves areas selected for the different measurements. The space between the two dark bars represents the area of 40 cm2 chosen for all measurements (see “Methods”). The sites in which the measurements of chlorophyll content index, chlorophyll fluorescence and gas exchange were performed are indicated by smaller open black circles, larger open blue circle and white rectangle circle inside the blue circle, respectively. [file 13007_2017_209_MOESM11_ESM.tif]

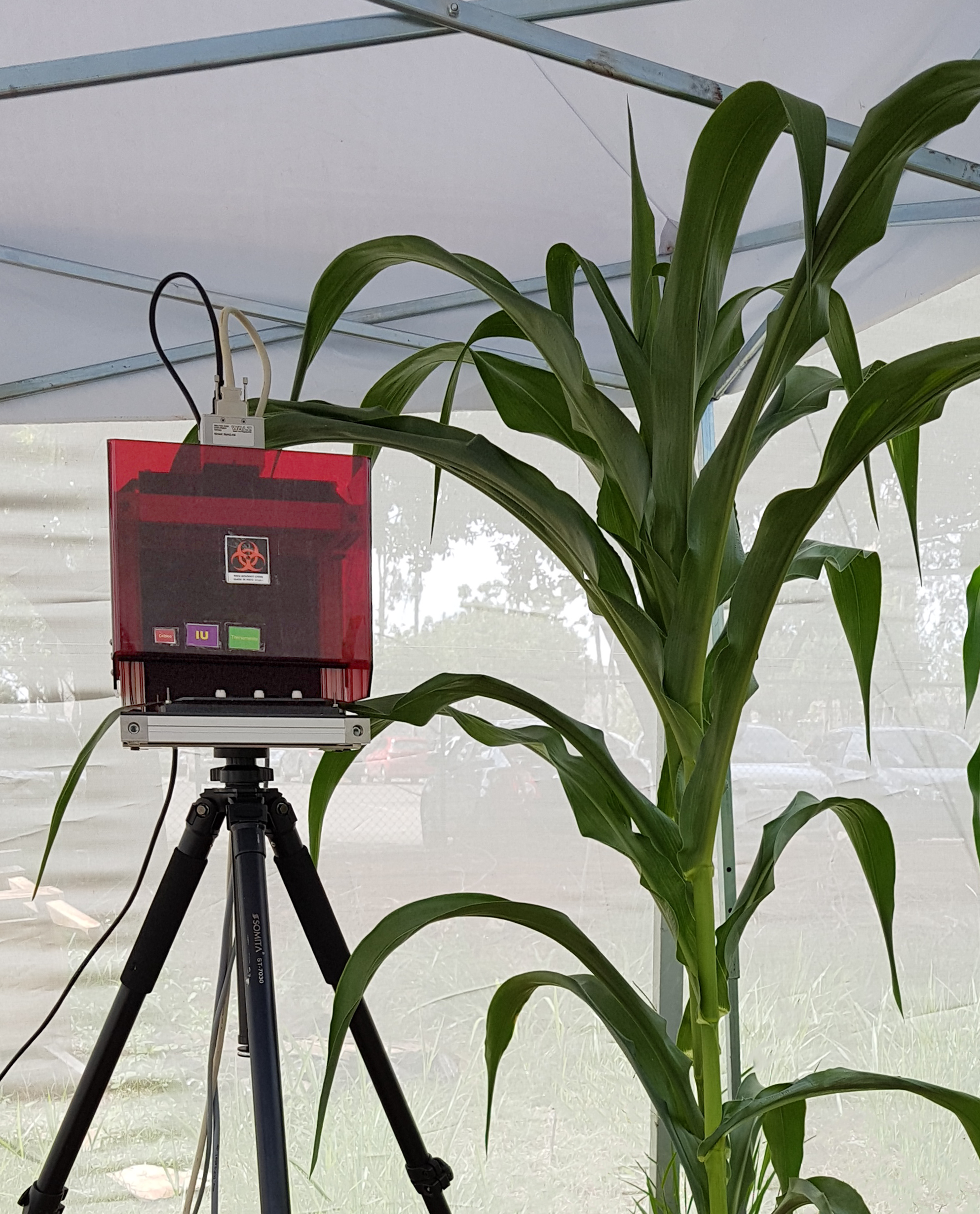

Supplement: Supplementary file 12 — Additional file 12. Digital image showing a maize plant being evaluated by IMAGING-PAM. For the measurement, an attached leaf was placed in the sample stage inside the measuring head which was closed and covered with a black fabric to prevent external light. [file 13007_2017_209_MOESM12_ESM.tif]
