# Supplementary material for: A procedure for maize genotypes discrimination to drought by chlorophyll fluorescence imaging rapid light curves
Source: Plant Methods. 2017 Jul 26;13:61. doi: 10.1186/s13007-017-0209-z (PMC5530575; doi:10.1186/s13007-017-0209-z)
Supplement: Supplementary file 2 — Additional file 2. (A) Resume of variance analyses from chlorophyll fluorescence parameters obtained by RLC’s in leaves of four the different maize genotypes grown under control or drought stress as a function of PAR. (B) Comparison of means by Tukey’s test (p < 0.05) from chlorophyll fluorescence parameters in the same RLC’s light step in leaves of maize genotypes grown under control or drought conditions. (C) Multi comparison of means by Tukey’s test (p < 0.05) from chlorophyll fluorescence parameters over the RLC’s light steps obtained in the leaves of maize genotypes continuously grown under soil water available at field capacity (control). (D) Multi comparison of means by Tukey’s test (p < 0.05) from chlorophyll fluorescence parameters over the RLC’s light steps obtained in leaves of maize genotypes in which the plants were subjected to water withholding (drought). [file 13007_2017_209_MOESM2_ESM.docx]

**Additional file 2**. A) Resume of variance analyses from chlorophyll fluorescence parameters obtained by RLC's in leaves of four the different maize genotypes grown under control or drought stress as a function of PAR. B) Comparison of means by Tukey's test (p<0.05) from chlorophyll fluorescence parameters in the same RLC's light step in leaves of maize genotypes grown under control or drought conditions. C) Multi comparison of means by Tukey's test (p<0.05) from chlorophyll fluorescence parameters over the RLC's light steps obtained in the leaves of maize genotypes continuously grown under soil water available at field capacity (control). D) Multi comparison of means by Tukey's test (p<0.05) from chlorophyll fluorescence parameters over the RLC's light steps obtained in leaves of maize genotypes in which the plants were subjected to water withholding (drought).

**Abbreviations**: soil water availability (SWA); photosynthetically active radiation (PAR); freedom of degree (f.d.); fluorescence yield (not necessarily in the steady-state) before application a saturate pulse (F); quantum yield of nonregulated energy dissipation [Y(NO)]; maximum fluorescence yield on light-adapted leaf (Fm'); effective PSII quantum yield [Y(II)]; coefficient of non-photochemical quenching (qN, lake model); quantum yield of regulated energy dissipation [Y(NPQ)]; non-photochemical quenching (NPQ); coefficient of photochemical quenching (qP, puddle model); coefficient of photochemical quenching (qL, lake model); apparent rate of photosynthesis (PS/50).

A) Resume of variance analyses

| **Source** | **f.d.** | **chlorophyll fluorescence parameter (mean squares)** | | | | |
| --- | --- | --- | --- | --- | --- | --- |
|  |  | **F** | **Y(NO)** | **Fm**' | **Y(II)** | **qN** |
| **Genotype** | 3 | 0.003392** | 0.002065* | 0.010589** | 0.006150** | 0.040622** |
| **SWA** | 1 | 0.010628** | 0.003127* | 0.226503** | 1.813908** | 2.249447** |
| **PAR** | 8 | 0.002374** | 0.023841** | 0.147185** | 1.451216** | 2.625222** |
| **Genotype x SWA** | 3 | 0.000072^ns^ | 0.024691** | 0.000803^ns^ | 0.039880** | 0.003241* |
| **Genotype x PAR** | 24 | 0.000186ns | 0.002932** | 0.000203ns | 0.003628** | 0.001049^ns^ |
| **SWA x PAR** | 8 | 0.002286** | 0.030590** | 0.002895** | 0.012371** | 0.076665** |
| **Genotype x SWA x PAR** | 24 | 0.000022^ns^ | 0.000607^ns^ | 0.000123^ns^ | 0.000649^ns^ | 0.000801^ns^ |
| **Error** | 288 | 0.000145 | 0.000744 | 0.000420 | 0.001212 | 0.001452 |
| **CV %** |  | 12.20 | 7.91 | 10.80 | 8.74 | 8.39 |

*^ns^, ^*^,^**^ not significantly different , significant at the 5% and 1% probability levels, respectively.*

A) Cont.

| **Source** | **f.d.** | **chlorophyll fluorescence parameter (mean squares)** | | | | |
| --- | --- | --- | --- | --- | --- | --- |
|  |  | **Y(NPQ)** | **NPQ** | **qP** | **qL** | **PS/50** |
| **Genotype** | 3 | 0.009950** | 0.012779** | 0.022352** | 0.061760** | 0.036495** |
| **SWA** | 1 | 1.664096** | 1.222551** | 2.568125** | 1.067982** | 5.969623** |
| **PAR** | 8 | 1.425347** | 0.941102** | 2.529512** | 3.029869** | 2.635782** |
| **Genotype x SWA** | 3 | 0.004469** | 0.004904** | 0.077015** | 0.058579** | 0.089983** |
| **Genotype x PAR** | 24 | 0.000542^ns^ | 0.000791^ns^ | 0.004409^ns^ | 0.003640^ns^ | 0.005724^ns^ |
| **SWA x PAR** | 8 | 0.059803** | 0.058921** | 0.072148** | 0.020022** | 0.359585** |
| **Genotype x SWA x PAR** | 24 | 0.000305^ns^ | 0.000318^ns^ | 0.002940^ns^ | 0.002642^ns^ | 0.004837^ns^ |
| **Error** | 288 | 0.000993 | 0.001171 | 0.003353 | 0.002850 | 0.005058 |
| **CV %** |  | 12.27 | 17.35 | 9.31 | 11.15 | 16.29 |

*^ns^, ^*^,^**^ not significantly different , significant at the 5% and 1% probability levels, respectively.*

B) Control x drought at the same PAR

| **PAR** | **SWA** | **chlorophyll fluorescence parameter (mean)** | | | | | | | | | |
| --- | --- | --- | --- | --- | --- | --- | --- | --- | --- | --- | --- |
|  |  | **F** | **Y(NO)** | **Fm'** | **Y(II)** | **qN** | **Y(NPQ)** | **NPQ** | **qP** | **qL** | **PS/50** |
| 0 | Drought | 0.09 a | 0.34 a | 0.26 b | 0.64 b | 0.07 a | 0.34 a | 0.01 a | 0.99 a | 0.96 a | 0.00 a |
|  | Control | 0.08 b | 0.26 b | 0.29 a | 0.72 a | 0.07 a | 0.26 b | 0.01 a | 0.93 b | 0.98 a | 0.00 a |
| 20 | Drought | 0.10 a | 0.38 a | 0.24 b | 0.55 b | 0.19 a | 0.38 a | 0.04 a | 0.90 b | 0.73 b | 0.09 a |
|  | Control | 0.09 b | 0.31 b | 0.28 a | 0.66 a | 0.11 b | 0.31 b | 0.02 a | 0.83 c | 0.78 a | 0.11 a |
| 55 | Drought | 0.10 a | 0.38 a | 0.21 b | 0.48 b | 0.33 a | 0.38 a | 0.08 a | 0.69 d | 0.62 b | 0.21 b |
|  | Control | 0.09 a | 0.32 b | 0.26 a | 0.64 a | 0.16 b | 0.32 b | 0.04 b | 0.57 e | 0.74 a | 0.29 a |
| 110 | Drought | 0.10 a | 0.37 a | 0.18 b | 0.38 b | 0.49 a | 0.37 a | 0.17 a | 0.52 ef | 0.47 b | 0.33 b |
|  | Control | 0.11 a | 0.35 b | 0.25 a | 0.58 a | 0.23 b | 0.35 b | 0.05 b | 0.48 fg | 0.62 a | 0.52 a |
| 185 | Drought | 0.10 b | 0.36 b | 0.14 b | 0.28 b | 0.67 a | 0.35 b | 0.27 a | 0.43 g | 0.34 b | 0.42 b |
|  | Control | 0.12 a | 0.39 a | 0.22 a | 0.46 a | 0.34 b | 0.39 a | 0.09 b | 0.98 a | 0.45 a | 0.72 a |
| 280 | Drought | 0.09 b | 0.34 b | 0.12 b | 0.19 b | 0.71 a | 0.34 b | 0.36 a | 0.99 a | 0.22 b | 0.44 b |
|  | Control | 0.12 a | 0.40 a | 0.19 a | 0.35 a | 0.49 b | 0.40 a | 0.16 b | 0.87 b | 0.34 a | 0.82 a |
| 335 | Drought | 0.09 b | 0.32 b | 0.11 b | 0.16 b | 0.76 a | 0.32 b | 0.41 a | 0.93 a | 0.18 b | 0.44 b |
|  | Control | 0.11 a | 0.37 a | 0.16 a | 0.31 a | 0.58 b | 0.37 a | 0.23 b | 0.78 b | 0.32 a | 0.86 a |
| 395 | Drought | 0.09 b | 0.31 b | 0.10 b | 0.13 b | 0.79 a | 0.31 b | 0.45 a | 0.90 a | 0.15 b | 0.42 b |
|  | Control | 0.10 a | 0.34 a | 0.14 a | 0.27 a | 0.66 b | 0.34 a | 0.29 b | 0.64 b | 0.29 a | 0.88 a |
| 460 | Drought | 0.08 b | 0.31 b | 0.09 b | 0.11 b | 0.81 a | 0.31 b | 0.49 a | 0.83 a | 0.13 b | 0.40 b |
|  | Control | 0.10 a | 0.32 a | 0.12 a | 0.23 a | 0.71 b | 0.32 a | 0.35 b | 0.49 b | 0.26 a | 0.88 a |

Means under the same PAR in a column followed by the same letter are not significantly different according to Tukey's test (P<0.05).

C) Control over PAR

| **PAR** | **chlorophyll fluorescence parameter (mean)** | | | | | | | | | |
| --- | --- | --- | --- | --- | --- | --- | --- | --- | --- | --- |
|  | **F** | **Y(NO)** | **Fm**' | **Y(II)** | **qN** | **Y(NPQ)** | **NPQ** | **qP** | **qL** | **PS/50** |
| 0 | 0.08 c | 0.26 f | 0.28 a | 0.72 a | 0.07 i | 0.01 g | 0.01 g | 0.99 a | 0.98 a | 0.00 f |
| 20 | 0.09 c | 0.31 e | 0.28 ab | 0.66 b | 0.12 h | 0.03 g | 0.02 fg | 0.93 b | 0.78 b | 0.11 e |
| 55 | 0.09 dc | 0.32 e | 0.26 bc | 0.64 b | 0.16 g | 0.05 fg | 0.04 fg | 0.90 b | 0.74 b | 0.29 d |
| 110 | 0.10 dc | 0.35 cd | 0.25 c | 0.57 c | 0.23 f | 0.08 f | 0.05 f | 0.83 c | 0.62 c | 0.53 c |
| 185 | 0.11 cb | 0.39 ab | 0.22 d | 0.46 d | 0.34 e | 0.14 e | 0.09 e | 0.69 d | 0.45 d | 0.72 b |
| 280 | 0.11 cb | 0.40 a | 0.19 e | 0.35 e | 0.49 d | 0.25 d | 0.16 d | 0.57 e | 0.34 e | 0.82 a |
| 335 | 0.11 ba | 0.37 bc | 0.16 f | 0.31 f | 0.59 c | 0.32 c | 0.23 c | 0.52 ef | 0.32 e | 0.86 a |
| 395 | 0.12 a | 0.34 cd | 0.14 fg | 0.26 g | 0.66 b | 0.39 b | 0.29 b | 0.48 fg | 0.30 ef | 0.88 a |
| 460 | 0.12 a | 0.33 de | 0.13 g | 0.23 h | 0.71 a | 0.44 a | 0.35 a | 0.43 g | 0.27 f | 0.88 a |

Means over PAR followed by the same letter in a column are not significantly different according to Tukey's test (P<0.05).

D) Drought over PAR

| **PAR** | **chlorophyll fluorescence parameter (mean)** | | | | | | | | | |
| --- | --- | --- | --- | --- | --- | --- | --- | --- | --- | --- |
|  | **F** | **Y(NO)** | **Fm**' | **Y(II)** | **qN** | **Y(NPQ)** | **NPQ** | **qP** | **qL** | **PS/50** |
| 0 | 0.09 bcd | 0.34 cde | 0.26 a | 0.61 a | 0.07 h | 0.02 i | 0.01 g | 0.98 a | 0.97 a | 0.00 e |
| 20 | 0.10 a | 0.39 a | 0.24 a | 0.55 b | 0.19 g | 0.06 h | 0.04 g | 0.87 b | 0.72 b | 0.09 d |
| 55 | 0.10 a | 0.38 ab | 0.21 b | 0.48 c | 0.340 f | 0.13 g | 0.08 f | 0.78 c | 0.62 c | 0.21 c |
| 110 | 0.10 ab | 0.37 ab | 0.18 c | 0.38 d | 0.49 e | 0.24 f | 0.17 e | 0.64 d | 0.47 d | 0.34 b |
| 185 | 0.09 abc | 0.36 bc | 0.15 d | 0.28 e | 0.62 d | 0.36 e | 0.27 d | 0.49 e | 0.34 e | 0.40 ab |
| 280 | 0.09 abcd | 0.34 cd | 0.12 e | 0.190 f | 0.71 c | 0.46 d | 0.36 c | 0.34 f | 0.22 f | 0.42 a |
| 335 | 0.08 bcd | 0.33 def | 0.11 ef | 0.16 g | 0.76 b | 0.51 c | 0.41 b | 0.28 fg | 0.18 fg | 0.43 a |
| 395 | 0.08 cd | 0.31 ef | 0.10 f | 0.13 gh | 0.79 ba | 0.55 b | 0.46 a | 0.25 gh | 0.16 g | 0.44 a |
| 460 | 0.08 d | 0.31 g | 0.09 f | 0.11 h | 0.810 a | 0.58 a | 0.49 a | 0.21 h | 0.13 g | 0.45 a |

Means over PAR followed by the same letter in a column are not significantly different according to Tukey's test (P<0.05).
