# Supplementary material for: A procedure for maize genotypes discrimination to drought by chlorophyll fluorescence imaging rapid light curves
Source: Plant Methods. 2017 Jul 26;13:61. doi: 10.1186/s13007-017-0209-z (PMC5530575; doi:10.1186/s13007-017-0209-z)
Supplement: Supplementary file 5 — Additional file 5. (A) Resume of variance analyses from gas exchange parameters obtained in leaves of four different maize genotypes grown under control or drought stress as a function of time. (B) Comparison of means by Tukey’s test (p < 0.05) from gas exchange parameters in leaves of maize genotypes grown under control or drought conditions. (C) Multi comparison of means by Tukey’s test (p < 0.05) from gas exchange parameters over time in the leaves of maize genotypes continuously grown under soil water available at field capacity (control). (D) Multi comparison of means by Tukey’s test (p < 0.05) from gas exchange parameters over time in the leaves of maize genotypes in which the plants were subjected to water withholding (drought). Both groups of plants (control and drought) were with soil water content at field capacity at the start of measurement (day 1). From there, the watering was withheld in the drought stressed plants until the substrate reached the theoretical wilting point (−1.5 MPa) on the 7th day. [file 13007_2017_209_MOESM5_ESM.docx]

**Additional file 5**. A) Resume of variance analyses from gas exchange parameters obtained in leaves of four different maize genotypes grown under control or drought stress as a function of time. B) Comparison of means by Tukey's test (p<0.05) from gas exchange parameters in leaves of maize genotypes grown under control or drought conditions. C) Multi comparison of means by Tukey's test (p<0.05) from gas exchange parameters over time in the leaves of maize genotypes continuously grown under soil water available at field capacity (control). D) Multi comparison of means by Tukey's test (p<0.05) from gas exchange parameters over time in the leaves of maize genotypes in which the plants were subjected to water withholding (drought). Both groups of plants (control and drought) were with soil water content at field capacity at the start of measurement (day 1). From there, the watering was withheld in the drought stressed plants until the substrate reached the theoretical wilting point (-1.5 MPa) on the 7^th^ day.

**Abbreviations**: soil water availability (SWA); freedom of degree (f.d.); net CO_2_ assimilation rate (*A*); stomatal conductance to water vapor (*gs*); intercellular CO_2_ concentration (*Ci*); transpiration rate (*E*).

A) Resume of variance analyses

| **Source** | **f.d.** | **gas exchange parameter (mean squares)** | | | |
| --- | --- | --- | --- | --- | --- |
|  |  | ***A*** | ***gs*** | ***Ci*** | ***E*** |
| **Time** | 3 | 5446.886829** | 0.777926** | 31678.226462** | 103.620727** |
| **Genotype** | 3 | 93.756726** | 0.024667* | 943.205837^ns^ | 1.112400^ns^ |
| **SWA** | 1 | 4695.693972** | 0.437751** | 42946.560056** | 58.424933** |
| **Time x Genotype** | 9 | 26.319331^ns^ | 0.011674^ns^ | 810.437214^ns^ | 0.590702** |
| **Time x SWA** | 3 | 2914.340009** | 0.206879** | 88860.155972** | 41.019568** |
| **Genotype x SWA** | 3 | 19.635121^ns^ | 0.009738^ns^ | 1432.050172^ns^ | 0.339586^ns^ |
| **Time x Genotype x SWA** | 9 | 13.036820^ns^ | 0.006675^ns^ | 333.299159^ns^ | 0.331484^ns^ |
| **Error** | 128 | 18.885308 | 0.007434 | 729.563256 | 0.423162 |
| **CV%** |  | 12.03 | 24.33 | 16.14 | 14.48 |

*^ns^, ^*^,^**^ not significantly different , significant at the 5% and 1% probability levels, respectively.*

B) Control x drought at the same time

| **Time (day)** | **SWA** | **gas exchange parameter (mean)** | | | |
| --- | --- | --- | --- | --- | --- |
|  |  | ***A*** | ***gs*** | ***Ci*** | ***E*** |
| **1** | Drought | 43.46 a | 0.41 a | 146.25 a | 5.30 a |
|  | Control | 44.47 a | 0.43 a | 151.26 a | 5.52 a |
| **3** | Drought | 44.64 a | 0.51 a | 169.22 a | 6.30 a |
|  | Control | 44.28 a | 0.46 a | 154.43 a | 6.10 a |
| **5** | Drought | 33.31 b | 0.27 b | 126.60 b | 3.39 b |
|  | Control | 39.96 a | 0.44 a | 174.02 a | 3.99 a |
| **7** | Drought | 01.46 b | 0.02 b | 293.04 a | 0.55 b |
|  | Control | 37.49 a | 0.29 a | 124.34 b | 4.76 a |

Means under the same time followed by the same letter in a column are not significantly different according to Tukey's test (p<0.05).

C) Control over time

| **Time (day)** | ***gas exchange parameter (mean)*** | | | |
| --- | --- | --- | --- | --- |
|  | ***A*** | ***gs*** | ***Ci*** | ***E*** |
| **1** | 44.47 a | 0.43 a | 151.26 b | 5.52 b |
| **3** | 44.28 a | 0.46 a | 154.43 b | 6.10 a |
| **5** | 39.96 b | 0.44 a | 174.02 a | 3.99 d |
| **7** | 37.49 b | 0.29 b | 124.34 c | 4.76 c |

Means over time followed by the same letter in a column are not significantly different according to Tukey's test (p<0.05).

D) Drought over time

| **Time (day)** | **gas exchange parameter (mean)** | | | |
| --- | --- | --- | --- | --- |
|  | ***A*** | ***gs*** | ***Ci*** | ***E*** |
| **1** | 43.46 a | 0.41 b | 146.25 c | 5.30 b |
| **3** | 44.64 a | 0.51 a | 169.22 b | 6.30 a |
| **5** | 33.31 b | 0.27 c | 126.60 d | 3.39 c |
| **7** | 01.46 c | 0.02 d | 293.04 a | 0.55 d |

Means over time followed by the same letter in a column are not significantly different according to Tukey's test (p<0.05).
