# Supplementary material for: A procedure for maize genotypes discrimination to drought by chlorophyll fluorescence imaging rapid light curves
Source: Plant Methods. 2017 Jul 26;13:61. doi: 10.1186/s13007-017-0209-z (PMC5530575; doi:10.1186/s13007-017-0209-z)
Supplement: Supplementary file 7 — Additional file 7. (A) Resume of variance analyses from chlorophyll fluorescence parameters obtained in leaves of four different maize genotypes grown under control or drought stress as a function of time. (B) Comparison of means by Tukey’s test (p < 0.05) from chlorophyll fluorescence parameters in leaves of maize genotypes grown under control or drought conditions. (C) Multi comparison of means by Tukey’s test (p < 0.05) from chlorophyll fluorescence parameters over time in the leaves of maize genotypes continuously grown under soil water available at field capacity (control). (D) Multi comparison of means by Tukey’s test (p < 0.05) from chlorophyll fluorescence parameters over time in the leaves of maize genotypes in which the plants were subjected to water withholding (drought). Only the data obtained at 280 µmol m−2 s−1 of actinic illumination were used. Both groups of plants (control and drought) were with soil water content at field capacity at the start of measurement (day 1). From there, the watering was withheld in the drought stressed plants until the substrate reached the theoretical wilting point (−1.5 MPa) on the 7th day. [file 13007_2017_209_MOESM7_ESM.docx]

**Additional file 7**. A) Resume of variance analyses from chlorophyll fluorescence parameters obtained in leaves of four different maize genotypes grown under control or drought stress as a function of time. B) Comparison of means by Tukey's test (p<0.05) from chlorophyll fluorescence parameters in leaves of maize genotypes grown under control or drought conditions. C) Multi comparison of means by Tukey's test (p<0.05) from chlorophyll fluorescence parameters over time in the leaves of maize genotypes continuously grown under soil water available at field capacity (control). D) Multi comparison of means by Tukey's test (p<0.05) from chlorophyll fluorescence parameters over time in the leaves of maize genotypes in which the plants were subjected to water withholding (drought). Only the data obtained at 280 µmol m^-2^ s^-1^ of actinic illumination were used. Both groups of plants (control and drought) were with soil water content at field capacity at the start of measurement (day 1). From there, the watering was withheld in the drought stressed plants until the substrate reached the theoretical wilting point (-1.5 MPa) on the 7^th^ day.

**Abbreviations**: soil water availability (SWA); freedom of degree (f.d.); fluorescence yield (not necessarily in the steady-state) before application a saturate pulse (F); quantum yield of nonregulated energy dissipation [Y(NO)]; maximum fluorescence yield on light-adapted leaf (Fm'); effective PSII quantum yield [Y(II)]; coefficient of non-photochemical quenching (qN, lake model); quantum yield of regulated energy dissipation [Y(NPQ)]; non-photochemical quenching (NPQ); coefficient of photochemical quenching (qP, puddle model); coefficient of photochemical quenching (qL, lake model); apparent rate of photosynthesis (PS/50).

A) Resume of variance analyses

| **Source** | **f.d.** | **chlorophyll fluorescence parameter (mean squares)** | | | | |
| --- | --- | --- | --- | --- | --- | --- |
|  |  | **F** | **Y(NO)** | **Fm**' | **Y(II)** | **qN** |
| **Time** | **3** | 0.002582** | 0.006763* | 0.029500** | 0.285749** | 0.236850** |
| **Genotype** | **3** | 0.000591^ns^ | 0.005886^ns^ | 0.000579^ns^ | 0.015398** | 0.006220^ns^ |
| **SWA** | **1** | 0.004884** | 0.001035* | 0.036603** | 0.385141** | 0.329332** |
| **Time x Genotype** | **9** | 0.000656* | 0.004661* | 0.000997^ns^ | 0.006822** | 0.006182ns |
| **Time x SWA** | **3** | 0.005465** | 0.008435* | 0.029273** | 0.250753** | 0.301884** |
| **Genotype x SWA** | **3** | 0.000181^ns^ | 0.004639^ns^ | 0.000062^ns^ | 0.005963* | 0.000178^ns^ |
| **Time x Genotype x SWA** | **9** | 0.000125^ns^ | 0.001423^ns^ | 0.000368^ns^ | 0.003705ns | 0.005342^ns^ |
| **Error** | **128** | 0.000329 | 0.002269 | 0.000693 | 0.002173 | 0.007801 |
| **CV%** | | 16.99 | 13.39 | 15.67 | 13.54 | 15.35 |

*^ns^, ^*^,^**^ not significantly different , significant at the 5% and 1% probability levels, respectively.*

1. Cont.

| **Source** | **fd** | **chlorophyll fluorescence parameter (mean squares)** | | | | |
| --- | --- | --- | --- | --- | --- | --- |
|  |  | **Y(NPQ)** | **NPQ** | **qP** | **qL** | **PS/50** |
| **Time** | **3** | 0.255613** | 0.202805** | 0.786869** | 0.357792** | 1.628399** |
| **Genotype** | **3** | 0.002449^ns^ | 0.000493^ns^ | 0.069082** | 0.079970** | 0.083694** |
| **SWA** | **1** | 0.346239** | 0.310024** | 1.030410** | 0.457318** | 2.512766** |
| **Time x Genotype** | **9** | 0.002377^ns^ | 0.003692ns | 0.028430** | 0.022471** | 0.035735** |
| **Time x SWA** | **3** | 0.261336 ** | 0.243996** | 0.741724** | 0.300090** | 1.483253** |
| **Genotype x SWA** | **3** | 0.000086^ns^ | 0.000814^ns^ | 0.008956^ns^ | 0.007870^ns^ | 0.040121* |
| **Time x Genotype x SWA** | **9** | 0.002474^ns^ | 0.003926^ns^ | 0.018787** | 0.012394^ns^ | 0.023730^ns^ |
| **Error** | **128** | 0.003685 | 0.004684 | 0.006491 | 0.007600 | 0.012948 |
| **CV%** | | 20.24 | 30.02 | 14.21 | 23.75 | 14.12 |

*^ns^, ^*^,^**^ not significantly different , significant at the 5% and 1% probability levels, respectively.*

1. Control x drought at the same time

| **Time (day)** | **SWA** | **chlorophyll fluorescence parameter (mean)** | | | | | | | | | |
| --- | --- | --- | --- | --- | --- | --- | --- | --- | --- | --- | --- |
|  |  | **F** | **Y(NO)** | **Fm**' | **Y(II)** | **qN** | **Y(NPQ)** | **NPQ** | **qP** | **qL** | **PS/50** |
| **1** | **Drought** | 0.12 a | 0.36 a | 0.19 a | 0.36 b | 0.55 a | 0.28 a | 0.19 a | 0.62 a | 0.39 a | 0.90 a |
|  | **Control** | 0.11 a | 0.33 a | 0.18 a | 0.38 a | 0.57 a | 0.28 a | 0.21 a | 0.66 a | 0.44 a | 0.94 a |
| **3** | **Drought** | 0.11 a | 0.38 a | 0.18 a | 0.41 a | 0.47 a | 0.21 a | 0.14 a | 0.65 a | 0.41 a | 0.92 a |
|  | **Control** | 0.11 a | 0.35 a | 0.18 a | 0.41 a | 0.50 a | 0.23 a | 0.17 a | 0.66 a | 0.42 a | 0.95 a |
| **5** | **Drought** | 0.10 a | 0.33 b | 0.15 b | 0.36 a | 0.61 a | 0.31 a | 0.26 a | 0.61 a | 0.40 a | 0.79 b |
|  | **Control** | 0.11 a | 0.36 a | 0.17 a | 0.38 a | 0.53 b | 0.25 b | 0.17 b | 0.63 a | 0.40 a | 0.89 a |
| **7** | **Drought** | 0.07 b | 0.37 a | 0.07 b | 0.05 b | 0.84 a | 0.57 a | 0.48 a | 0.07 b | 0.04 b | 0.09 b |
|  | **Control** | 0.12 a | 0.36 a | 0.18 a | 0.38 a | 0.51 b | 0.24 b | 0.17 b | 0.64 a | 0.40 a | 0.92 a |

Means under the same time in a column followed by the same letter are not significantly different according to Tukey's test (p<0.05).

1. Control over time

| **Time (day)** | **chlorophyll fluorescence parameter (mean)** | | | | | | | | | |
| --- | --- | --- | --- | --- | --- | --- | --- | --- | --- | --- |
|  | **F** | **Y(NO)** | **Fm**' | **Y(II)** | **qN** | **Y(NPQ)** | **NPQ** | **qP** | **qL** | **PS/50** |
| **1** | 0.11 a | 0.33 a | 0.18 a | 0.38 a | 0.57 a | 0.28 a | 0.21 a | 0.66 a | 0.44 a | 0.94 a |
| **3** | 0.11 a | 0.35 a | 0.18 a | 0.41 a | 0.50 a | 0.23 a | 0.17 a | 0.66 a | 0.42 a | 0.95 a |
| **5** | 0.11 a | 0.36 a | 0.17 a | 0.38 a | 0.53 a | 0.25 a | 0.17 a | 0.63 a | 0.40 a | 0.89 a |
| **7** | 0.12 a | 0.36 a | 0.18 a | 0.38 a | 0.51 a | 0.24 a | 0.17 a | 0.64 a | 0.40 a | 0.92 a |

Means over time in a column followed by the same letter are not significantly different according to Tukey's test (p<0.05).

1. Drought over time

| **Time (day)** | **chlorophyll fluorescence parameter (mean)** | | | | | | | | | |
| --- | --- | --- | --- | --- | --- | --- | --- | --- | --- | --- |
|  | **F** | **Y(NO)** | **Fm**' | **Y(II)** | **qN** | **Y(NPQ)** | **NPQ** | **qP** | **qL** | **PS/50** |
| **1** | 0.12 a | 0.36 a | 0.19 a | 0.36 b | 0.55 c | 0.28 b | 0.19 c | 0.62 a | 0.39 a | 0.90 a |
| **3** | 0.11 a | 0.38 a | 0.18 a | 0.41 a | 0.47 d | 0.21 c | 0.14 d | 0.65 a | 0.41 a | 0.92 a |
| **5** | 0.09 c | 0.33 b | 0.15 b | 0.36 b | 0.61 b | 0.31 b | 0.26 b | 0.61 a | 0.40 a | 0.79 b |
| **7** | 0.07 b | 0.37 a | 0.07c | 0.05 c | 0.84 a | 0.57 a | 0.48 a | 0.07 b | 0.04 b | 0.09 c |

Means over time in a column followed by the same letter are not significantly different according to Tukey's test (p<0.05).
