# Supplementary material for: A procedure for maize genotypes discrimination to drought by chlorophyll fluorescence imaging rapid light curves
Source: Plant Methods. 2017 Jul 26;13:61. doi: 10.1186/s13007-017-0209-z (PMC5530575; doi:10.1186/s13007-017-0209-z)
Supplement: Supplementary file 8 — Additional file 8. (A) Resume of variance analyses from chlorophyll fluorescence parameters obtained by RLC’s applied to the leaves of different maize plants grown under control or drought conditions obtained at the 7th day after water withholding. Only the data obtained at 280 µmol m−2 s−1 of actinic illumination were used. (B) Comparison of means by Tukey’s test (p < 0.05) from chlorophyll fluorescence parameters in the leaves of the same maize genotype grown under control or drought conditions. (C) Comparison of means by Tukey’s test (p < 0.05) from chlorophyll fluorescence parameters in the leaves of different maize genotypes continuously grown under soil water available at field capacity (control). (D) Comparison of means by Tukey’s test (p < 0.05) from chlorophyll fluorescence parameters in the leaves of different maize genotypes in which the plants were subjected to water withholding (drought). [file 13007_2017_209_MOESM8_ESM.docx]

**Additional file 8**. A) Resume of variance analyses from chlorophyll fluorescence parameters obtained by RLC's applied to the leaves of different maize plants grown under control or drought conditions obtained at the 7^th^ day after water withholding. Only the data obtained at 280 µmol m^-2^ s^-1^ of actinic illumination were used. B) Comparison of means by Tukey's test (p<0.05) from chlorophyll fluorescence parameters in the leaves of the same maize genotype grown under control or drought conditions. C) Comparison of means by Tukey's test (p<0.05) from chlorophyll fluorescence parameters in the leaves of different maize genotypes continuously grown under soil water available at field capacity (control). D) Comparison of means by Tukey's test (p<0.05) from chlorophyll fluorescence parameters in the leaves of different maize genotypes in which the plants were subjected to water withholding (drought).

**Abbreviations:** soil water availability (SWA); freedom of degree (f.d.); Fluorescence yield (not necessarily in the steady-state) before application a saturate pulse (F); Quantum yield of nonregulated energy dissipation (YNO); Maximum fluorescence yield on light-adapted leaf (Fm'); Effective PSII quantum yield (YII); Coefficient of non-photochemical quenching (qN, lake model); Quantum yield of regulated energy dissipation (YNPQ); Non-photochemical quenching (NPQ); Coefficient of photochemical quenching (qP, puddle model); Coefficient of photochemical quenching (qL, lake model); Apparent rate of photosynthesis (PS/50).

A) Resume of variance analyses

| **Source** | **f.d.** | **chlorophyll fluorescence parameter (mean squares)** | | | | |
| --- | --- | --- | --- | --- | --- | --- |
|  |  | **F** | **Y(NO)** | **Fm**' | **Y(II)** | **qN** |
| **Genotype** | 3 | 0.001157** | 0.011451** | 0.000537* | 0.013030** | 0.007774** |
| **SWA** | 1 | 0.019272** | 0.000265^ns^ | 0.118266** | 1.121580** | 1.138725** |
| **Genotype x SWA** | 3 | 0.000298^ns^ | 0.007159* | 0.000133^ns^ | 0.011476** | 0.005229** |
| **Error** | 32 | 0.000233 | 0.002119 | 0.000153 | 0.000718 | 0.000965 |
| **CV%** |  | 15.63 | 12.53 | 9.46 | 12.17 | 4.59 |

*^ns^, ^*^,^**^ not significantly different , significant at the 5% and 1% probability levels, respectively.*

A) Cont.

| **Source** | **f.d.** | **chlorophyll fluorescence parameter (mean squares) Continuation** | | | | |
| --- | --- | --- | --- | --- | --- | --- |
|  |  | **Y(NPQ)** | **NPQ** | **qP** | **qL** | **PS/50** |
| **Genotype** | 3 | 0.000330^ns^ | 0.001523^ns^ | 0.077252** | 0.047717** | 0.046427** |
| **SWA** | 1 | 1.087351** | 0.966277** | 3.231354** | 1.331885** | 6.834329** |
| **Genotype x SWA** | 3 | 0.002500^ns^ | 0.003766^ns^ | 0.048689** | 0.026144** | 0.056490** |
| **Error** | 32 | 0.001709 | 0.001927 | 0.001784 | 0.002523 | 0.001318 |
| **CV%** |  | 10.03 | 13.41 | 11.81 | 22.27 | 7.12 |

*^ns^, ^*^,^**^ not significantly different , significant at the 5% and 1% probability levels, respectively.*

B) Comparison of control x drought at the same genotpe

| **Genotype** | **SWA** | **chlorophyll fluorescence parameter (mean)** | | | | | | | | | |
| --- | --- | --- | --- | --- | --- | --- | --- | --- | --- | --- | --- |
|  |  | **F** | **Y(NO)** | **Fm**' | **Y(II)** | **qN** | **Y(NPQ)** | **NPQ** | **qP** | **qL** | **PS/50** |
| **BRS 1030** | Drought | 0.07 b | 0.31 a | 0.08 b | 0.13 b | 0.83 a | 0.55 a | 0.49 a | 0.29 b | 0.17 b | 0.25 b |
|  | Control | 0.13 a | 0.32 a | 0.18 a | 0.41 a | 0.54 b | 0.26 b | 0.19 b | 0.67 a | 0.45 a | 0.95 a |
| **BRS 1010** | Drought | 0.07 b | 0.40 b | 0.07 b | 0.02 b | 0.87 a | 0.58 a | 0.46 a | 0.00 b | 0.00 b | 0.00 b |
|  | Control | 0.09 a | 0.33 a | 0.17 a | 0.41 a | 0.56 b | 0.25 b | 0.19 b | 0.71 a | 0.50 a | 1.00 a |
| **DKB 390** | Drought | 0.07 b | 0.39 a | 0.07 b | 0.00 b | 0.85 a | 0.60 a | 0.49 a | 0.00 b | 0.00 b | 0.00 b |
|  | Control | 0.12 a | 0.37 a | 0.19 a | 0.38 a | 0.46 b | 0.24 b | 0.14 b | 0.57 a | 0.38 a | 0.87 a |
| **2B 707** | Drought | 0.09 b | 0.36 a | 0.08 b | 0.05 b | 0.83 a | 0.57 a | 0.48 a | 0.00 b | 0.00 b | 0.12 b |
|  | Control | 0.13 a | 0.42 a | 0.19 a | 0.33 a | 0.47 b | 0.24 b | 0.15 b | 0.60 a | 0.29 a | 0.85 a |

Means of the same maize genotype in a column followed by the same letter are not significantly different at according to Tukey's test (P<0.05).

C) Multi comparison among genotypes under control

| **Genotype** | **chlorophyll fluorescence parameter (mean)** | | | | | | | | | |
| --- | --- | --- | --- | --- | --- | --- | --- | --- | --- | --- |
|  | **F** | **Y(NO)** | **Fm**' | **Y(II)** | **qN** | **Y(NPQ)** | **NPQ** | **qP** | **qL** | **PS/50** |
| **BRS 1030** | 0.13 a | 0.32 b | 0.18 ab | 0.41 a | 0.54 a | 0.26 a | 0.19 a | 0.67 ab | 0.45 ab | 0.95 b |
| **BRS 1010** | 0.09 b | 0.33 b | 0.17 b | 0.41 a | 0.56 a | 0.25 a | 0.19 a | 0.71 a | 0.50 a | 1.00 a |
| **DKB 390** | 0.12 ab | 0.37 ab | 0.19 ab | 0.38 a | 0.46 b | 0.24 a | 0.14 a | 0.57 c | 0.38 b | 0.87 c |
| **2B 707** | 0.13 a | 0.42 a | 0.19 a | 0.33 b | 0.47 b | 0.24 a | 0.15 a | 0.60 bc | 0.29 c | 0.85 c |

Means followed by the same letter in a column are not significantly different at according to Tukey's test (P<0.05).

D) Multi comparison among genotypes under drought

| **Genotype** | **chlorophyll fluorescence parameter (mean)** | | | | | | | | | |
| --- | --- | --- | --- | --- | --- | --- | --- | --- | --- | --- |
|  | **F** | **Y(NO)** | **Fm**' | **Y(II)** | **qN** | **Y(NPQ)** | **NPQ** | **qP** | **qL** | **PS/50** |
| **BRS 1030** | 0.07 a | 0.31 b | 0.08 a | 0.13 a | 0.83 a | 0.55 a | 0.49 a | 0.29 a | 0.17 a | 0.25 a |
| **BRS 1010** | 0.07 a | 0.40 a | 0.07 a | 0.02 bc | 0.87 a | 0.58 a | 0.46 a | 0.00 b | 0.00 b | 0.00 c |
| **DKB 390** | 0.07 a | 0.39 a | 0.07 a | 0.00 c | 0.85 a | 0.60 a | 0.49 a | 0.00 b | 0.00 b | 0.00 c |
| **2B 707** | 0.09 a | 0.36 ab | 0.08 a | 0.05 b | 0.83 a | 0.57 a | 0.48 a | 0.00 b | 0.00 b | 0.12 b |

Means followed by the same letter in a column are not significantly different at according to Tukey's test (P<0.05).
